# Airborne DNA reveals predictable spatial and seasonal dynamics of fungi

**DOI:** 10.1038/s41586-024-07658-9

**Published:** 2024-07-10

**Authors:** Nerea Abrego, Brendan Furneaux, Bess Hardwick, Panu Somervuo, Isabella Palorinne, Carlos A. Aguilar-Trigueros, Nigel R. Andrew, Ulyana V. Babiy, Tan Bao, Gisela Bazzano, Svetlana N. Bondarchuk, Timothy C. Bonebrake, Georgina L. Brennan, Syndonia Bret-Harte, Claus Bässler, Luciano Cagnolo, Erin K. Cameron, Elodie Chapurlat, Simon Creer, Luigi P. D’Acqui, Natasha de Vere, Marie-Laure Desprez-Loustau, Michel A. K. Dongmo, Ida B. Dyrholm Jacobsen, Brian L. Fisher, Miguel Flores de Jesus, Gregory S. Gilbert, Gareth W. Griffith, Anna A. Gritsuk, Andrin Gross, Håkan Grudd, Panu Halme, Rachid Hanna, Jannik Hansen, Lars Holst Hansen, Apollon D. M. T. Hegbe, Sarah Hill, Ian D. Hogg, Jenni Hultman, Kevin D. Hyde, Nicole A. Hynson, Natalia Ivanova, Petteri Karisto, Deirdre Kerdraon, Anastasia Knorre, Irmgard Krisai-Greilhuber, Juri Kurhinen, Masha Kuzmina, Nicolas Lecomte, Erin Lecomte, Viviana Loaiza, Erik Lundin, Alexander Meire, Armin Mešić, Otto Miettinen, Norman Monkhouse, Peter Mortimer, Jörg Müller, R. Henrik Nilsson, Puani Yannick C. Nonti, Jenni Nordén, Björn Nordén, Veera Norros, Claudia Paz, Petri Pellikka, Danilo Pereira, Geoff Petch, Juha-Matti Pitkänen, Flavius Popa, Caitlin Potter, Jenna Purhonen, Sanna Pätsi, Abdullah Rafiq, Dimby Raharinjanahary, Niklas Rakos, Achala R. Rathnayaka, Katrine Raundrup, Yury A. Rebriev, Jouko Rikkinen, Hanna M. K. Rogers, Andrey Rogovsky, Yuri Rozhkov, Kadri Runnel, Annika Saarto, Anton Savchenko, Markus Schlegel, Niels Martin Schmidt, Sebastian Seibold, Carsten Skjøth, Elisa Stengel, Svetlana V. Sutyrina, Ilkka Syvänperä, Leho Tedersoo, Jebidiah Timm, Laura Tipton, Hirokazu Toju, Maria Uscka-Perzanowska, Michelle van der Bank, F. Herman van der Bank, Bryan Vandenbrink, Stefano Ventura, Solvi R. Vignisson, Xiaoyang Wang, Wolfgang W. Weisser, Subodini N. Wijesinghe, S. Joseph Wright, Chunyan Yang, Nourou S. Yorou, Amanda Young, Douglas W. Yu, Evgeny V. Zakharov, Paul D. N. Hebert, Tomas Roslin, Otso Ovaskainen

**Affiliations:** 1 Department of Biological and Environmental Science, University of Jyväskylä, Jyväskylä, Finland; 2 Department of Agricultural Sciences, University of Helsinki, Helsinki, Finland; 3 Organismal and Evolutionary Biology Research Programme, Faculty of Biological and Environmental Sciences, University of Helsinki, Helsinki, Finland; 4 Natural History Museum, University of New England, Armidale, New South Wales Australia; 5 Faculty of Science and Engineering, Southern Cross University, Northern Rivers, New South Wales Australia; 6 Wrangel Island State Nature Reserve, Pevek, Russia; 7 Department of Biological Sciences, MacEwan University, Edmonton, Alberta Canada; 8 Centro de Zoología Aplicada, Facultad de Ciencias Exactas Físicas y Naturales, Universidad Nacional de Córdoba, Córdoba, Argentina; 9 Sikhote-Alin State Nature Biosphere Reserve named after K. G. Abramov, Terney, Russia; 10 School of Biological Sciences, The University of Hong Kong, Hong Kong SAR, China; 11 Institute of Marine Sciences, Consejo Superior de Investigaciones Científicas (CSIC), Passeig Marítim de la Barceloneta, Barcelona, Spain; 12 Institute of Arctic Biology, University of Alaska, Fairbanks, AK USA; 13 Department of Conservation Biology, Institute for Ecology, Evolution and Diversity, Faculty of Biological Sciences, Goethe-University Frankfurt, Frankfurt am Main, Germany; 14 Bavarian Forest National Park, Grafenau, Germany; 15 Bayreuth Center of Ecology and Environmental Research (BayCEER), University of Bayreuth, Bayreuth, Germany; 16 Instituto Multidisciplinario de Biología Vegetal, Consejo Nacional de Investigaciones Científicas y Técnicas (CONICET), Córdoba, Argentina; 17 Department of Environmental Science, Saint Mary’s University, Halifax, Nova Scotia Canada; 18 Department of Ecology, Swedish University of Agricultural Sciences (SLU), Uppsala, Sweden; 19 Molecular Ecology and Evolution at Bangor (MEEB), School of Biological Sciences, Bangor University, Bangor, Wales; 20 Research Institute on Terrestrial Ecosystems - IRET, National Research Council - CNR and National Biodiversity Future Center, Palermo, Italy; 21 Natural History Museum of Denmark, University of Copenhagen, Copenhagen, Denmark; 22 BIOGECO, INRAE, University of Bordeaux, Cestas, France; 23 International Institute of Tropical Agriculture (IITA), Yaoundé, Cameroon; 24 Greenland Institute of Natural Resources, Nuuk, Greenland; 25 Department of Entomology, California Academy of Sciences, San Francisco, CA USA; 26 Madagascar Biodiversity Center, Parc Botanique et Zoologique de Tsimbazaza, Antananarivo, Madagascar; 27 Legado das Águas, Reserva Votorantin, Tapiraí, Brazil; 28 Department of Environmental Studies, University of California Santa Cruz, Santa Cruz, CA USA; 29 Department of Life Sciences, Aberystwyth University, Aberystwyth, UK; 30 Biodiversity and Conservation Biology Research Unit, SwissFungi Data Center, Swiss Federal Research Institute WSL, Birmensdorf, Switzerland; 31 Swedish Polar Research Secretariat, Abisko Scientific Research Station, Abisko, Sweden; 32 Center for Tropical Research, Congo Basin Institute, University of California Los Angeles, Los Angeles, CA USA; 33 Department of Ecoscience, Aarhus University, Roskilde, Denmark; 34 Research Unit in Tropical Mycology and Plant–Soil Fungi Interactions, Faculty of Agronomy, University of Parakou, Parakou, Republic of Benin; 35 Canadian High Arctic Research Station, Polar Knowledge Canada, Cambridge Bay, Nunavut Canada; 36 Department of Integrative Biology, College of Biological Science, University of Guelph, Guelph, Ontario Canada; 37 School of Science, University of Waikato, Hamilton, New Zealand; 38 Department of Microbiology, University of Helsinki, Helsinki, Finland; 39 Natural Resources Institute Finland (Luke), Helsinki, Finland; 40 Center of Excellence in Fungal Research, Mae Fah Luang University, Chiang Rai, Thailand; 41 Pacific Biosciences Research Center, University of Hawaii at Manoa, Honolulu, HI USA; 42 Centre for Biodiversity Genomics, University of Guelph, Guelph, Ontario Canada; 43 Nature Metrics North America Ltd., Guelph, Ontario Canada; 44 Plant Pathology Group, Institute of Integrative Biology, ETH Zurich, Zurich, Switzerland; 45 Plant Health, Natural Resources Institute Finland (Luke), Jokioinen, Finland; 46 Science Department, National Park Krasnoyarsk Stolby, Krasnoyarsk, Russia; 47 Institute of Ecology and Geography, Siberian Federal University, Krasnoyarsk, Russia; 48 Department of Botany and Biodiversity Research, University of Vienna, Vienna, Austria; 49 Centre d’Études Nordiques and Canada Research Chair in Polar and Boreal Ecology, Department of Biology, Université de Moncton, Moncton, New Brunswick Canada; 50 Department of Evolutionary Biology and Environmental Sciences, University of Zürich, Zurich, Switzerland; 51 Laboratory for Biological Diversity, Rudjer Boskovic Institute, Zagreb, Croatia; 52 Finnish Museum of Natural History, University of Helsinki, Helsinki, Finland; 53 Centre for Mountain Futures, Kunming Institute of Botany, Chinese Academy of Sciences, Kunming, China; 54 Department of Conservation Biology and Forest Ecology, Julius Maximilians University Würzburg, Rauhenebrach, Germany; 55 Department of Biological and Environmental Sciences, Gothenburg Global Biodiversity Centre, University of Gothenburg, Gothenburg, Sweden; 56 Norwegian Institute for Nature Research (NINA), Oslo, Norway; 57 Nature Solutions, Finnish Environment Institute (Syke), Helsinki, Finland; 58 Department of Biodiversity, Institute of Biosciences, São Paulo State University, Rio Claro, Brazil; 59 Department of Entomology and Acarology, Laboratory of Pathology and Microbial Control, University of São Paulo, Piracicaba, Brazil; 60 Department of Geosciences and Geography, Faculty of Science, University of Helsinki, Helsinki, Finland; 61 State Key Laboratory for Information Engineering in Surveying, Mapping and Remote Sensing, Wuhan University, Wuhan, China; 62 Wangari Maathai Institute for Environmental and Peace Studies, University of Nairobi, Kangemi, Kenya; 63 Laboratory of Biochemistry, Wageningen University, Wageningen, the Netherlands; 64 School of Science and the Environment, University of Worcester, Worcester, UK; 65 Department of Ecosystem Monitoring, Research & Conservation, Black Forest National Park, Bad Peterstal-Griesbach, Germany; 66 School of Resource Wisdom, University of Jyväskylä, Jyväskylä, Finland; 67 Biodiversity Unit, University of Turku, Turku, Finland; 68 School of Science, Mae Fah Luang University, Chiang Rai, Thailand; 69 Southern Scientific Center of the Russian Academy of Sciences, Rostov-on-Don, Russia; 70 State Nature Reserve Olekminsky, Olekminsk, Russia; 71 Mycology and Microbiology Center, University of Tartu, Tartu, Estonia; 72 Institute of Ecology and Earth Sciences, University of Tartu, Tartu, Estonia; 73 Arctic Research Center, Aarhus University, Roskilde, Denmark; 74 Forest Zoology, TUD Dresden University of Technology, Berchtesgaden, Germany; 75 Terrestrial Ecology Research Group, Department of Life Science Systems, School of Life Sciences, Technical University of Munich, Freising, Germany; 76 Department of Environmental Science, Aarhus University, Roskilde, Denmark; 77 Field Station Fabrikschleichach, Department of Animal Ecology and Tropical Biology (Zoology III), Julius Maximilians University Würzburg, Rauhenebrach, Germany; 78 Kevo Subarctic Research Institute, Biodiversity Unit, University of Turku, Utsjoki, Finland; 79 College of Science, King Saud University, Riyadh, Saudi Arabia; 80 School of Natural Science and Mathematics, Chaminade University of Honolulu, Honolulu, HI USA; 81 Laboratory of Ecosystems and Coevolution, Graduate School of Biostudies, Kyoto University, Kyoto, Japan; 82 Center for Living Systems Information Science (CeLiSIS), Graduate School of Biostudies, Kyoto University, Kyoto, Japan; 83 African Centre for DNA Barcoding (ACDB), University of Johannesburg, Auckland Park, South Africa; 84 Sudurnes Science and Learning Center, Sandgerði, Iceland; 85 State Key Laboratory of Genetic Resources and Evolution, Kunming Institute of Zoology, Chinese Academy of Sciences, Kunming, China; 86 Smithsonian Tropical Research Institute, Balboa, Panama; 87 School of Biological Sciences, University of East Anglia, Norwich, UK; 88 Yunnan Key Laboratory of Biodiversity and Ecological Security of Gaoligong Mountain, Kunming Institute of Zoology, Center of Excellence in Animal Evolution and Genetics, Chinese Academy of Sciences, Kunming, China; 89 Centre for Biodiversity Dynamics, Department of Biology, Norwegian University of Science and Technology, Trondheim, Norway

**Keywords:** Biodiversity, Biogeography, Community ecology, Ecology

## Abstract

Fungi are among the most diverse and ecologically important kingdoms in life. However, the distributional ranges of fungi remain largely unknown as do the ecological mechanisms that shape their distributions^1,2^. To provide an integrated view of the spatial and seasonal dynamics of fungi, we implemented a globally distributed standardized aerial sampling of fungal spores^3^. The vast majority of operational taxonomic units were detected within only one climatic zone, and the spatiotemporal patterns of species richness and community composition were mostly explained by annual mean air temperature. Tropical regions hosted the highest fungal diversity except for lichenized, ericoid mycorrhizal and ectomycorrhizal fungi, which reached their peak diversity in temperate regions. The sensitivity in climatic responses was associated with phylogenetic relatedness, suggesting that large-scale distributions of some fungal groups are partially constrained by their ancestral niche. There was a strong phylogenetic signal in seasonal sensitivity, suggesting that some groups of fungi have retained their ancestral trait of sporulating for only a short period. Overall, our results show that the hyperdiverse kingdom of fungi follows globally highly predictable spatial and temporal dynamics, with seasonality in both species richness and community composition increasing with latitude. Our study reports patterns resembling those described for other major groups of organisms, thus making a major contribution to the long-standing debate on whether organisms with a microbial lifestyle follow the global biodiversity paradigms known for macroorganisms^4,5^.

## Abstract (web summary)

Using a globally distributed standardized aerial sampling of fungal spores, we show that the hyperdiverse kingdom of fungi follows globally highly predictable spatial and temporal dynamics, with seasonality in both species richness and community composition increasing with latitude.

## Main

Global biodiversity of microorganisms and the factors determining their distribution and activity remain poorly known despite their major ecological and economic importance in various ecosystems^6–8^. Recently developed technologies and analytical methods provide groundbreaking opportunities for both the improved sampling of biodiversity and unravelling how biodiversity is structured at large spatial and temporal scales^9–11^. These new methods thus provide the opportunity to uncover previously unmapped biodiversity patterns of microbial communities and to discover the ecological processes that shape their diversity at the global scale.

Fungi are among the most diverse and ecologically important living organisms. They mediate crucial processes in terrestrial ecosystems as decomposers of dead tissues (saprotrophs), mutualistic partners (ectomycorrhizal, ericoid, endophytic and lichenized fungi) and as pathogens of almost all terrestrial multicellular organisms. In spite of its importance, fungal diversity remains poorly explored^1^. Although roughly 156,000 species of fungi have been scientifically described and recognized as valid to date^12^, estimates of global species richness vary from 0.5 to 10 million^13,14^. Consequently the global spatial and temporal distributions of fungi remain largely unknown. Recently developed DNA-based survey methods have greatly improved our knowledge of large-scale patterns of fungal diversity^15–19^. Soil sampling has proved particularly popular, driven by an interest in the key functions of soil fungi as plant symbionts and nutrient cyclers^2,16,18,20^. Nevertheless it remains to be seen whether patterns in soil-borne fungi reflect patterns in other fungal taxa, or indeed in general biodiversity^21^. In fact, studies targeting different fungal groups have produced disparate results. Tedersoo et al.^16^ found that, although overall fungal diversity in soil increases toward the Equator, this pattern does not apply to ectomycorrhizal fungi, which are most diverse in boreal and temperate regions. However, a meta-analysis of metabarcoding data from soil- and root-associated fungi reported that total fungal diversity is higher at higher latitudes^19^. Among further disparities, the diversity of leaf-associated aquatic fungi has been found to peak at mid-latitudes^22^ whereas that of terrestrial leaf endophytes increases in tropical regions^23^.

Local studies conducted in both Arctic and temperate environments have shown that fungal activity presents pronounced seasonal variation^24–28^ whereas a study conducted in the tropics showed no such variation^29^, suggesting that seasonality may be latitude dependent. However, most large-scale surveys of fungi have included limited temporal replication of the same locations, leaving a major knowledge gap about their global seasonal dynamics. The few larger-scale studies that involve temporal replication include meta-analyses on heterogeneous datasets^30,31^ or historic records of fruiting-body occurrences^32^. The general conclusion drawn from these studies is that the composition and biomass of fungal communities follow the phenology of their hosts and seasonal changes in precipitation and temperature. Hence, the lack of controlling for effects of local seasonal variation may have also confounded some conclusions on the global spatial patterning of fungal diversity.

**Fig. 1:**
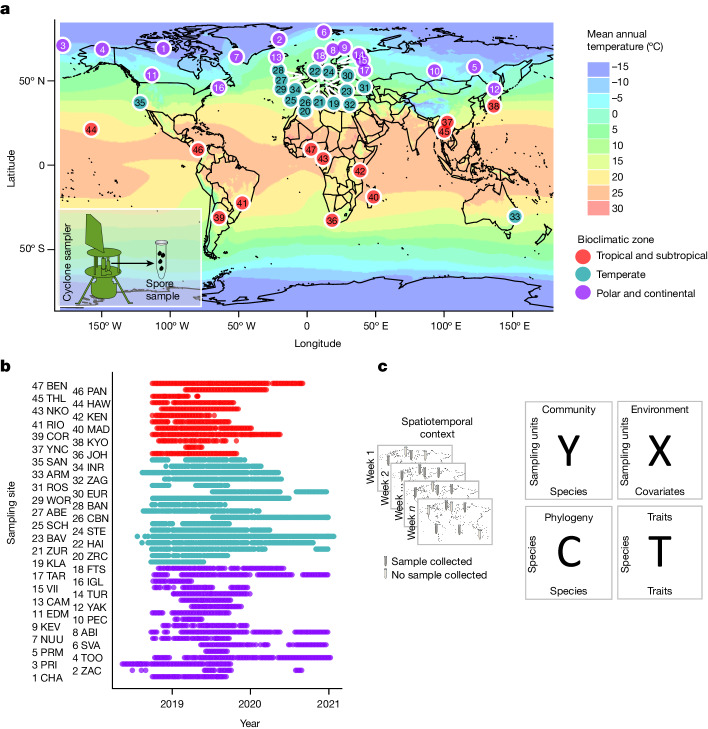
GSSP study design and data. **a**, The sampling sites include locations in the tropical-subtropical (red), temperate (cyan) and polar-continental (purple) climatic zones, shown here superimposed on a map of MAT. Airborne fungal samples were collected by a cyclone sampler, each sample consisting of fungal spores filtered from 24 m^3^ of air during the 24 h sampling period. **b**, The study design included weekly samples taken over 1–2 years, with some variation among sites due to logistical constraints. The site name abbreviations (three-letter codes next to the site numbers) correspond to those used in the published data^59^. **c**, The data-generation pipeline produced data matrices that were used for the ecological analyses: the spatial and temporal coordinates of the samples, species occurrence data (Y), climatic and weather data (X), fungal guild and spore size data (T) and taxonomic affiliations serve as a proxy for phylogenetic relationships (C).

A recent methodological breakthrough regarding the surveying of fungi consists of sampling fungal spores (and other airborne particles, which may include fungal structures such as hyphae and soredia) from the air, followed by DNA sequencing and sequence-based species identification^33^. Air sampling has shown higher diversity and stronger ecological signals in community composition than soil sampling^34^. The feasibility of air sampling to investigate global patterns of fungal diversity was recently demonstrated^35^. Because this method captures airborne fungal spores, it depicts reproduction and dispersal at high temporal resolution. Here we report on the application of air sampling for fungal spores in a new initiative called the Global Spore Sampling Project (GSSP)^3^. The GSSP involves 47 sampling locations distributed across all continents except Antarctica, each location collecting two 24 h samples per week over 1 year or more (Fig. 1a,b). Although the European temperate region is overrepresented in the data, the sampling locations also include Arctic, temperate and tropical areas from other regions (Fig. 1a). As described in detail in ref. ^3^, we targeted DNA sequencing to a part of the nuclear ribosomal internal transcribed spacer (ITS) region, which is the universal molecular barcode for fungi^36^. However, we note that for some fungal taxa other markers are better suited, such as the nuclear small subunit ribosomal RNA gene fragment for arbuscular mycorrhiza^37^. We applied a DNA spike-in to generate quantitative estimates of change in the amount of DNA^35^. To convert sequence data to species data we denoised the former to form amplicon sequence variants (ASVs)^38^, applied probabilistic taxonomic placement using Protax^39,40^ and used constrained dynamic clustering to group these ASVs into species-level operational taxonomic units (OTUs)^41^. These OTUs were then classified into previously known versus unknown taxa at all taxonomic levels from phylum to species^3^. To link spatiotemporal patterns in species composition to the ecological drivers behind them, we complement here the fungal species data derived from DNA analyses with environmental and trait data (Fig. 1c). Trait data were compiled using guild and spore size data from several sources (Methods), and environmental data include time- and site-specific climatic data from the Copernicus Climate Change Service Climate Data Store^42^.

The fully standardized sampling of fungi at unprecedented spatial and temporal scales enabled an integrated analysis of the ecological drivers behind the spatial and seasonal patterns of global fungal diversity. To achieve this, we first examined how fungal communities differ among the major bioclimatic zones and the extent to which climatic variables explain such differences. We expected to find a clear differentiation in community composition among the main bioclimatic zones, although we expected the spatial differentiation of airborne spores to be less pronounced than previously reported in soil-based studies^16,19^ because microscopic propagules can be expected to mix more readily in air (although samples were collected close to the ground, and often within habitats with limited air flow compared with open areas). Second, we examined how global seasonal patterns of airborne fungi vary with latitude and weather conditions. We expected higher levels of seasonality in species richness and amount of fungal DNA towards higher latitudes, where resources are available for shorter periods of time and where local weather conditions may have a stronger effect on reproductive phenology^32^. Finally we examined whether the ecological drivers shaping the composition of fungal communities translate into predictable variation in species-level traits. To this end we asked whether species’ responses to climatic and seasonal factors are phylogenetically and functionally structured. As relevant traits we considered fungal guild^16,43^ and spore size^44,45^. We expected to find higher seasonality in host-dependent guilds (pathogenic and symbiotic fungi) than in free-living guilds (saprotrophs), but that spatial patterns of seasonality should be consistent across guilds. We expected to find predictable seasonal variation in spore size, reflecting taxonomic turnover throughout the seasons. Finally, because earlier research has found phylogenetic niche conservatism reflected in the large-scale biogeography of soil fungi^46^, we expected to find a phylogenetic signal on the responses of air-fungal communities to the environmental factors that influence their large-scale distributions.

## Climatic effects on spatial distribution

**Fig. 2:**
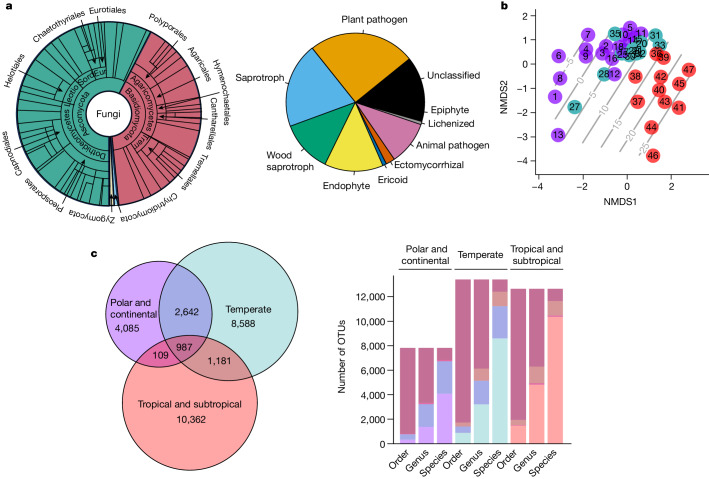
Taxonomic, functional and spatial variation in airborne fungal diversity. **a**, Taxonomic and functional guild composition of the data as weighted by prevalence (that is, the number of samples from which the taxon was found). Taxonomic composition is shown for the levels of phylum, class and order. Trophic guild composition is shown based on ref. ^54^. **b**, Variation in the composition of the fungal community among sites illustrated in the NMDS ordination space, with contour lines representing the MAT (°C) of the site. **c**, Venn diagram showing the number of OTUs that were distinct or shared among the three major climatic zones included in our study. Note that shown are raw numbers that do not control for the somewhat smaller sampling effort in the tropical-subtropical zone (Fig. 1b). The bar chart shows the number of OTUs that belonged to a genus or order that was either distinct or shared among the three climatic zones. Note that the species-level bars replicate the patterns shown in the Venn diagram.

Our samples of airborne fungi include all major taxonomic groups (Fig. 2a). However, some fungal groups are overrepresented and others underrepresented as compared with previously reported patterns among soil fungi. The air samples are particularly rich in plant pathogens, general saprotrophs and wood saprotrophs whereas other common groups such as ectomycorrhizal and lichenized fungi are relatively poorly represented (Fig. 2a).

Among the 27,954 species-level OTUs detected in this study, only 3.5% were observed in all three climatic zones (Fig. 2c). As expected, sampling locations in the polar-continental zone shared the fewest species with sampling locations in the tropical-subtropical zone. However, most order-level taxa were present in all three climatic zones (Fig. 2c). Such an increase in taxonomic overlap among regions with increasing taxonomic rank is also reflected by the stability of the proportions of species belonging to different phyla, with the proportion of Ascomycota spp. being 55–59% and that of Basidiomycota spp. being 38–43% within each of the three climatic zones.

Among the ten most prevalent genera in our data (Extended Data Table 1), seven belonged to the phylum Ascomycota (out of which four belonged to the order Pleosporales) and three to Basidiomycota (out of which two to the order Tremellales). Overall, the three most prevalent genera were the ascomycetes *Cladosporium*, *Ascochyta* and *Alternaria*. Genera included in the list of the ten most prevalent genera in all three climatic zones were the ascomycetes *Cladosporium*, *Ascochyta, Alternaria* and *Aureobasidium* and the basidiomycete *Cryptococcus*.

**Extended Data Fig. 1:**
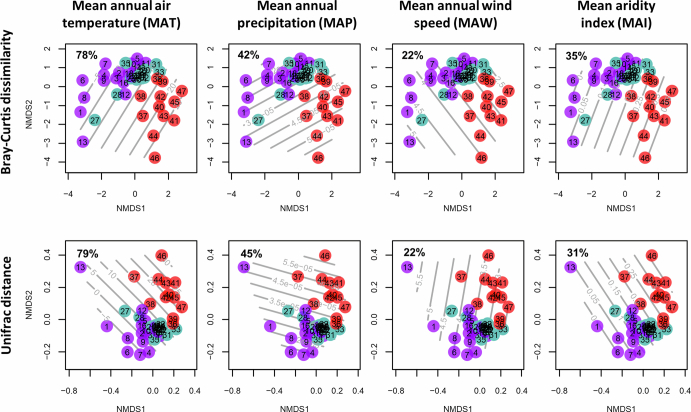
Results of ordination analyses. The upper panels show the results for the Bray-Curtis dissimilarity, and the lower panels for the Unifrac distance. The bolded percentages show the proportion of deviance in the ordination space explained by the focal environmental covariate: MAT (1^st^ column of panels), MAP (2^nd^ column of panels), MAW (3^rd^ column of panels), or MAI (4^th^ column of panels).

Species composition of local fungal communities was most strongly affected by the mean annual air temperature (MAT) of the site, which, when used as the sole environmental predictor, explained 78% of the deviance in ordination space (Fig. 2b and Extended Data Fig. 1). By comparison, mean annual precipitation (MAP) at the site explained 42% and the mean aridity index (MAI) 35%, whereas mean annual wind speed—which could have added to the mixing of spores to the atmosphere—did not explain much of the deviance (22%). We then compared the relative importance of differences in MAT (selecting for species with similar environmental preferences) and differences in space (probably reflecting the potential for dispersal between two sites, as well as other environmental conditions not considered in the analyses). Because spatial and environmental distances were correlated, we disentangled the effects of these by partitioning variance in community dissimilarity. We found the direct contribution of spatial distance to be 12%, that of climatic distance (derived from MAT) to be 7% and their shared contribution to be 22%. When repeating the analyses with climatic distances derived from MAP (or MAI), the direct contribution of spatial distance was 29% (27%), that of climatic distance 2% (0%) and their shared contribution to be 6% (7%). Hence MAT, rather than MAP or MAI, turned out to be a key driver in determining the large-scale distributions of airborne fungi.

## Seasonal patterns and weather responses

**Fig. 3:**
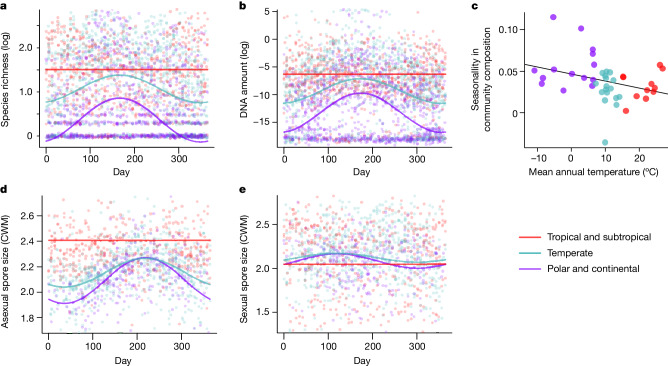
Seasonal variation in airborne fungal diversity. **a**–**e**, The lines representing species richness (**a**), DNA amount (**b**), community composition (**c**) and CWM of asexual (**d**) and sexual (**e**) spore size show the predictions of the best-supported linear mixed models (Methods) for tropical-subtropical (red), temperate (cyan) and polar-continental (purple) climatic zones. Note that the predictions are shown for the Northern Hemisphere whereas for the Southern Hemisphere the seasonal patterns would be mirror images. For community composition (**c**), seasonality for each site is defined as the difference in Jaccard index between samples taken in the same season versus those taken in different seasons (Methods). **a**,**b**,**d**,**e**, The dots representing the raw data have been slightly jittered to show overlap. The line in **c** shows that seasonality in community composition was higher at colder sites (linear regression, *P* = 0.04).

Within airborne spore communities, both OTU diversity and DNA amount increased towards the Equator (Fig. 3a,b). This result was robust with respect to seasonality, because tropical-subtropical sites hosted a greater diversity of fungal species and greater amounts of DNA than temperate and polar-continental sites at all times of the year (Fig. 3a,b). In terms of temporal patterns, seasonal variation in both DNA amount and species richness increased as expected with distance from the Equator, being highest in the Arctic (Fig. 3a,b). During winter at the polar-continental sites, few air samples had detectable levels of fungal DNA and the amount of DNA and number of species both showed a sharp peak during the growing season (Fig. 3a,b). In samples from temperate sites, fungal DNA was found throughout the year but its amount increased markedly from spring to autumn, with the lowest values in winter. In tropical-subtropical sites, fungal DNA amount was high throughout the year. The composition of the fungal community followed the same pattern: in polar-continental sites there was greater turnover in species composition from spring to autumn than in tropical regions during a comparable period (Fig. 3c). However, a comparison of linear mixed models fitted to the data on DNA amount and species richness (Supplementary Information) showed that, although the effect of seasonality generally increased with latitude, the exact timing and amplitude of seasonal variation also had a site-specific component. Thus, although we found that the phenology of fungal spore production is largely consistent within each latitudinal zone, the site-specific component suggests that local factors also play a role in controlling the timing of sporulation. Regarding the effects of weather, we found that both the amount of DNA and observed species richness were generally higher for warm and windy sampling days (Supplementary Information). Whereas most trophic guilds followed the same pattern as overall species richness, endophytes and lichenized species showed higher richness on days with little precipitation. These results were consistent across all latitudes in the sense that, for all but one response variable, the best-supported model was that of constant weather effects (model W1; Methods).

## Phylogenetic and functional structure

**Extended Data Fig. 2:**
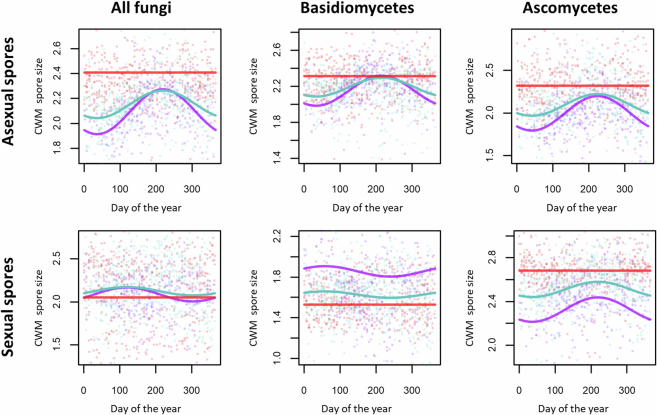
Seasonal variation in community weighted mean spore size. The lines in the panels show the predictions of the best-supported linear mixed models (see Methods) for tropical-subtropical (red), temperate (cyan), and polar-continental (purple) climatic zones. Note that the predictions are shown for the Northern Hemisphere, whereas for the Southern Hemisphere the seasonal patterns would be mirror images. The response variable in each panel is the community weighted mean spore size for the type of spores shown in the row and column titles. The dots that show the raw data have been slightly jittered to reveal overlap.

The proportion of fungal occurrences for which we had at least family-level information about asexual (respectively, sexual) spore volume varied between 72 and 74% (respectively, 68–70%) among the three climatic zones. However, species-level information was more frequent in the polar-continental and temperate zones (7–8% for asexual and 12–13% for sexual spores) than in the tropical zone (8% for asexual and 5% for sexual spores). Assuming that the detected species were in the asexual stage, these were largest in the tropical-subtropical zone whereas, assuming that the spores were in the sexual stage, these were largest in the temperate and polar-continental zones (Fig. 3d,e). In temperate and polar-continental zones, spore sizes showed marked seasonality, the mean asexual spore size peaking in the autumn and the mean sexual spore size in spring (Fig. 3d,e). This difference between asexual and sexual spores prevailed across all species and within Basidiomycota, but not within Ascomycota (Supplementary Information and Extended Data Fig. 2).

**Extended Data Fig. 3:**
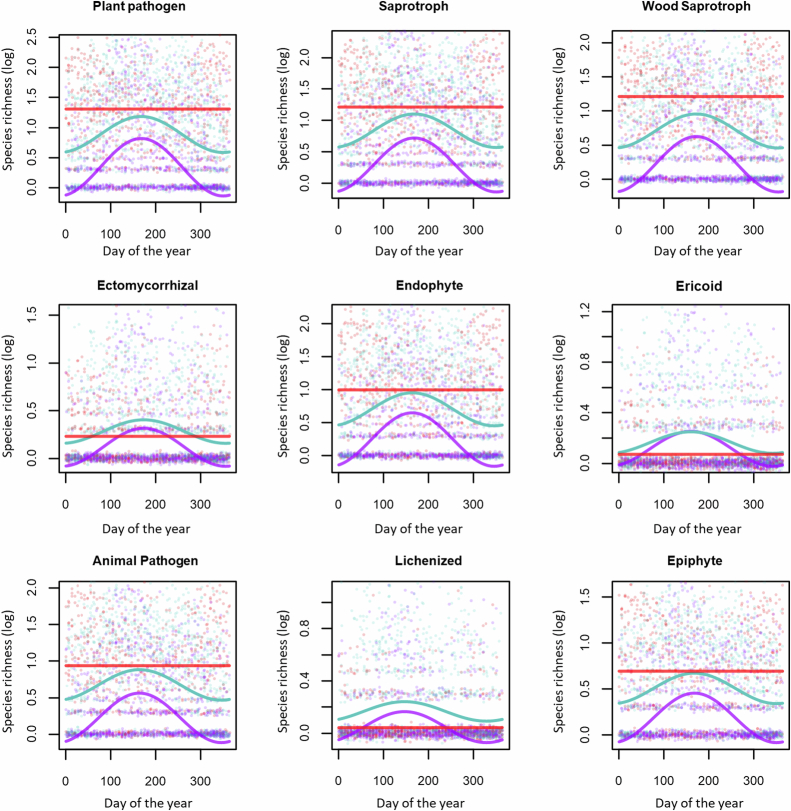
Seasonal variation in airborne fungal diversity of different trophic guilds. The lines in the panels show the predictions of the best-supported linear mixed models (see Methods) for tropical-subtropical (red), temperate (cyan), and polar-continental (purple) climatic zones. Note that the predictions are shown for the Northern Hemisphere, whereas for the Southern Hemisphere the seasonal patterns would be mirror images. The response variable in each panel is the species richness of the trophic guild shown indicated on top of the panels. The dots that show the raw data have been slightly jittered to reveal overlap.

Following the main patterns found for total fungal species richness, all fungal guilds exhibited strong seasonality in species richness in the polar-continental and temperate zones (Supplementary Information and Extended Data Fig. 3). Most guilds were more abundant in the tropics even during the peak season, with the exceptions of ericoid mycorrhizal, ectomycorrhizal and lichenized fungi, which were most abundant in the temperate region (Extended Data Fig. 3).

**Fig. 4:**
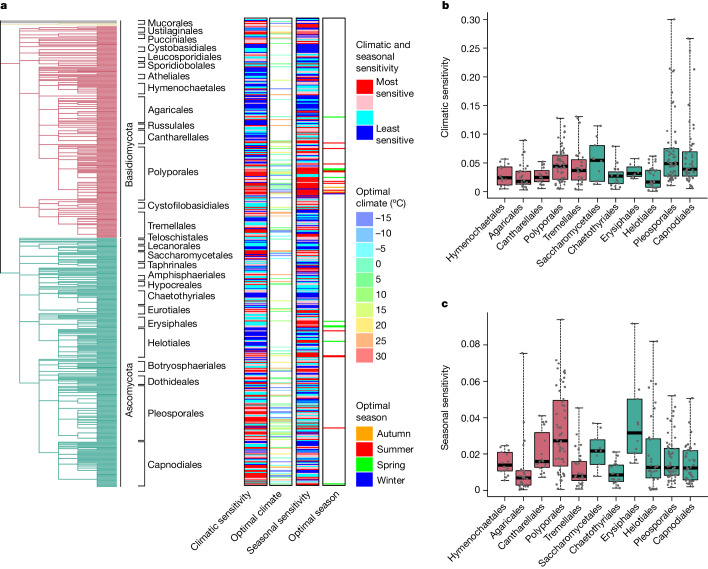
Phylogenetic signal in climatic and seasonal variation. **a**–**c**, All results are based on a joint-species distribution model fitted to the 485 most common species. **a**, Quantification of variation in climatic sensitivity, optimal climate, seasonality sensitivity and optimal season among species. For climatic and seasonal sensitivity the colours show the proportion of variance explained by the second-order polynomial of the MAT of that site (for climatic sensitivity) and by the periodic functions of sin(2π*d*/365) and cos(2π*d*/365), where *d* represents the Julian day of the year (for seasonal sensitivity), coded as blue, cyan, pink and red for the four quartiles. For optimal climate we show the MAT at which the second-order polynomial of that MAT was maximized (that is, the point at which a further increase in MAT will change an estimated increase to an estimated decrease in species occurrence) in the colour scale of the world map shown in Fig. 1a. For optimal season we show the season at which the estimated occurrence of the species will peak, with colours coded as blue for winter (December–February in the Northern Hemisphere; for the Southern Hemisphere we assumed a 6 month difference in seasonality), green for spring (March–May), red for summer (June–August) and orange for autumn (September–November). Cases in which climatic or seasonal sensitivities were too low to determine the optimal climate or season are shown in white. **b**,**c**, Boxplots show the distributions of climatic (**b**) and seasonal sensitivities (**c**) for those orders represented in these analyses by at least ten species. Lines show the medians, boxes the lower and upper quartiles and whiskers the minimum and maximum values. The raw data are shown by dots that have been jittered to show overlapping points. For the list of taxa included in the analysis, see Supplementary Information.

To determine how the phylogenetic relatedness of fungal species affects global distribution and sporulation patterns, we performed Hierarchical Modelling of Species Communities (HMSC) analysis^47^ in which we used as a proxy for the phylogenetic tree a taxonomy of OTUs at the levels of kingdom, phylum, class, order, family, genus and species^3^. Even if this model included only MAT and seasonality as predictors, it reached a high explanatory power (averaged over species, mean area under the curve = 0.90, mean Tjur’s *R*^2^ = 0.16). This analysis showed variation in the strength of phylogenetic signal among how species responded to focal environmental predictors. Among the species-level responses to environmental conditions, climatic sensitivity showed a moderate phylogenetic signal (Pagel’s λ = 0.28, *P* = 4 × 10^−12^), as illustrated by groups of highly related species that showed high or low climatic sensitivity (red and blue bands, respectively, in Fig. 4a in the climatic sensitivity column)—for example, the orders Agaricales and Helotiales being little influenced by climate (Fig. 4b). By contrast, the optimal MAT of the site at which the probability of species occurrence is predicted to be maximized did not show any phylogenetic signal (Pagel’s λ = −0.01, *P* = 0.81). Thus some species within the same group preferred colder temperatures whereas others preferred warmer temperatures (Fig. 4a). When we measured the seasonal sensitivity of species by the proportion of variation in species occurrence explained by latitude-dependent seasonality, we observed a strong phylogenetic signal (Pagel’s λ = 0.39, *P* = 2 × 10^−16^). In particular, species within the orders Polyporales and Erysiphales showed pronounced seasonal dynamics whereas the orders Agaricales, Tremellales and Chaetothyriales showed low sensitivity to seasonality (Fig. 4c). Regarding the timing of the seasonal peak, we did not observe any phylogenetic signal (Pagel’s λ = −0.04, *P* = 0.80). However, this lack of a signal may be partially explained by the fact that few species showed sufficient seasonality for the time of the optimal season to be defined (Fig. 4a).

## Discussion

Our results show that fungi follow predictable latitudinal diversity gradients that resemble other major groups of organisms^48^. This finding represents a major contribution to the long-standing debate over whether organisms with a microbial lifestyle follow the global biodiversity paradigms known for macroorganisms^4,5^. Our results are consistent with an increasing body of literature showing that, like macroorganisms, microbial communities are spatially structured at large scales^6,7,16^. Interestingly, only a small minority of all species-level OTUs detected in our study were observed in all three climatic zones. These widespread species were Ascomycota genera that have previously been found to be very common in both soil^49^ and air^17^. However, the vast majority of OTUs were detected only within one climatic zone and the spatiotemporal patterns of species richness and community composition were highly constrained by climatic conditions. Although previous large-scale studies of soil fungi have found clear effects of climate on community composition^16,19^, the fact that in our data MAT explains most of the variation in the distributions of fungi is striking, especially given that our data are based on the dispersal stage of airborne spores. Likewise, previous studies on soil fungal communities have found that biomes, as defined based on MAT and MAP, explain a major part of their global distribution^16^.

A major advantage of our data is the high level of temporal replication, enabling a global analysis of climatic effects on the phenology of fungal reproduction. Seasonality in both the amount of DNA and species richness of airborne fungi increased with increasing distance from the Equator and therefore seasonality was highest in Arctic climates. Less trivially, we found that seasonal turnover in community composition increased with increasing distance from the Equator, even if tropical regions also show high seasonality (for example, rainy versus dry periods). In line with this finding, a long-term study of airborne fungi in the tropics showed no seasonality^29^. In addition to seasonal effects, our study also highlights the importance of short-term local weather conditions on the diversity or sporulation phenology of airborne fungi. The results showed that airborne fungal species richness peaks during warm and windy sampling days, a finding coinciding with previous observations that temperature influences fungal reproductive phenology^32^ and that spore release peaks when wind speeds are high^50^.

Comparison of trophic guilds showed that not only overall species richness, but also most guilds, were most abundant in the tropics, with the notable exceptions of lichenized, ericoid mycorrhizal and ectomycorrhizal species. This result is in line with the patterns demonstrated for soil fungi by Tedersoo et al.^16^, who also found a general increase towards the tropics, except for ectomycorrhizal fungi which were most diverse in boreal and temperate regions. Whereas the higher diversity of these fungal groups at higher latitudes could be related to greater knowledge gaps of their diversity in the tropics, this result could also reflect the distribution and diversity of their host species^51^. To minimize the possibility of misleading artefact due to knowledge gaps, we borrowed information among taxonomic levels for the functional classifications, making a compromise between minimization of bias (by inclusion of not only the minority of OTUs reliably classified to species but also genus- or family-level classifications) and minimization of the noise of false classifications (by not borrowing information from ranks higher than family). In terms of seasonality, many earlier studies have reported longer sporulation and reproductive seasons in warmer regions for specific parts of the world and for particular groups of fungi^32,52^. Our results generalize these earlier findings to the global distribution of the entire fungal kingdom: all fungal guilds showed consistent and predictable patterns, with sporulation activity being shorter and more pronounced towards higher latitudes. Regarding spore size, we found that asexual spore size decreased but sexual spore size increased with increasing distance from the Equator. During the main reproductive season in the temperate and polar-continental zones, we further found asexual spore size to increase but sexual spore size to decrease during the season. The latter result, which is consistent with the earlier finding of Kauserud et al.^53^, is partially generated by ascomycetes having on average larger sexual spores^54^ and earlier sporulation phenology than basidiomycetes^33^. Our study reports opposing spatial and temporal patterns between sexual and asexual spores, suggesting contrasting evolutionary forces behind the size of these two types of dispersal propagule. This result may also relate to the opposing environmental triggers of sexual and asexual spore production, with the former occurring especially under unfavourable environmental conditions such as at the end of the growing season^55,56^.

In terms of the processes that structure ecological communities, we may distinguish between (1) the ultimate evolutionary processes that give rise to species and determine their traits and (2) the proximate contemporary ecological processes that shape the assembly of communities^57,58^. Our data on global aerial communities shed light on both aspects. In terms of evolutionary processes, fungi exhibited a strong niche conservatism regarding sensitivity to dispersal seasonality and moderate conservatism for sensitivity to climatic conditions. These results suggest that fungi have continuously adapted to climatic conditions rather than being stuck in their ancestral climatic niches. This interpretation is supported by the fact that whereas most species showed climatically restricted distributions, the majority of genera and the vast majority of orders were detected in all three climatic zones. The high phylogenetic signal in dispersal seasonality was driven by certain taxonomic groups. In particular, Polyporales showed a high level of seasonality for almost all species. Our findings suggest that Polyporales have been especially adapted to seasonal climates, possibly because their morphological and physiological traits support high spore production for a brief portion of the fruiting season. Among the ecological selection processes,we showed that environmental drivers, in particular MAT, play a major structuring role in fungal communities at large scales.

Whereas substrate-specific sampling will mainly show the DNA of mycelia locally present in the focal substrates, aerial DNA will provide an integrated view of airborne propagules from all substrates. As evidence, all trophic guilds supported by the guild database we used are represented in the data. However, some functional groups were better represented than others, highlighting the importance of surveying different complementary substrates to gain a complete view of fungal diversity. Importantly, the proportional representation of aerial fungal taxa is clearly affected by their dispersal strategy: in particular, plant pathogens, saprotrophs and wood saprotrophs were very abundant in our data (Fig. 2a). By contrast, ectomycorrhizal fungi, not all of which produce conspicuous and abundant above-ground reproductive bodies, contributed only a small fraction of airborne spores globally (Fig. 2a). This points to other dispersal means—for example, via mycophagous animals—as being important for this functional group. Alternatively, the relative scarcity of airborne spores from ectomycorrhizal fungi may be due to the trade-off between spore size and number^45^. Because many ectomycorrhizal fungi develop large spores they are expected to produce fewer spores, which in turn would appear less frequently in airborne data. Note that typically both large and small spores are unicellular and contain a single nucleus.

Our results demonstrate that the sampling of airborne DNA can provide a synthetic, cumulative view of global fungal diversity across individual substrates. This integrated view provides a huge step forward in the understanding of the distributions and dynamics of the whole fungal kingdom, which has lagged behind research in other major organism groups partially due to methodological difficulties in surveying fungi comprehensively. Overall our results show highly predictable patterns of spatial and seasonal variation in airborne fungi and suggest that the drivers of microbial community assembly are largely similar to those determining the assembly of macroorganisms. Our results highlight the role of temperature as an underlying driver of fungal dynamics, with fungal diversity increasing with warmer climates and sporulation activity increasing with warmer days. This finding suggests that global climate change with generally warming climates will have a major role in restructuring fungal communities.

## Methods

### Sampling, sequencing and bioinformatics

For full details on study design and sample collection, DNA extraction and sequencing, bioinformatic processing, as well as technical data validation, see ref. ^3^. Here we summarize these steps.

The study design consists of 47 sampling sites, each equipped with a cyclone sampler (Burkard Cyclone Sampler for Field Operation, Burkard Manufacturing Co Ltd; http://burkard.co.uk/product/cyclone-sampler-for-field-operation). The sampling sites were selected to represent local natural environments in which intensive, continuous sampling was possible. The cyclone samplers collected particles of greater than 1 µm in size from the air directly into a sterile Eppendorf vial, with average air throughput of 23.8 m^3^ during each 24 h sampling period. Before the start of our global sampling, a field test was performed to evaluate the quantity of fungal DNA collected over different time frames. We also included field blanks handled with and without gloves, in which the sampler was not activated, and the Eppendorf vials were removed after 1 min and sealed. As a result of the field tests we selected a 24 h sampling period and instructed the participants to handle samples with gloves and to clean the cyclone parts monthly.

**Extended Data Fig. 4:**
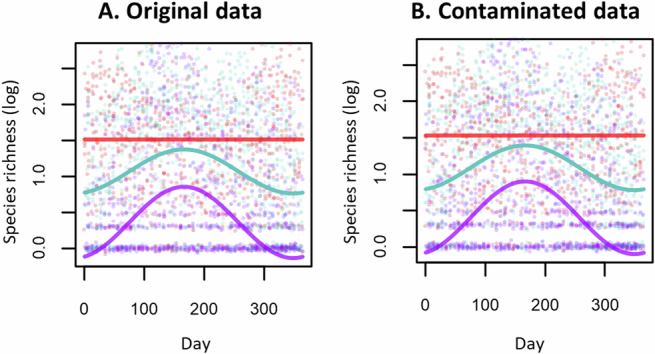
Comparison species richness analyses with original data (A; replicated from Fig. 3a of the main document) and data contaminated with sequences counts observed in negative controls (B). The results are shown for one of the ten replicates of the contaminated datasets.

**Extended Data Fig. 5:**
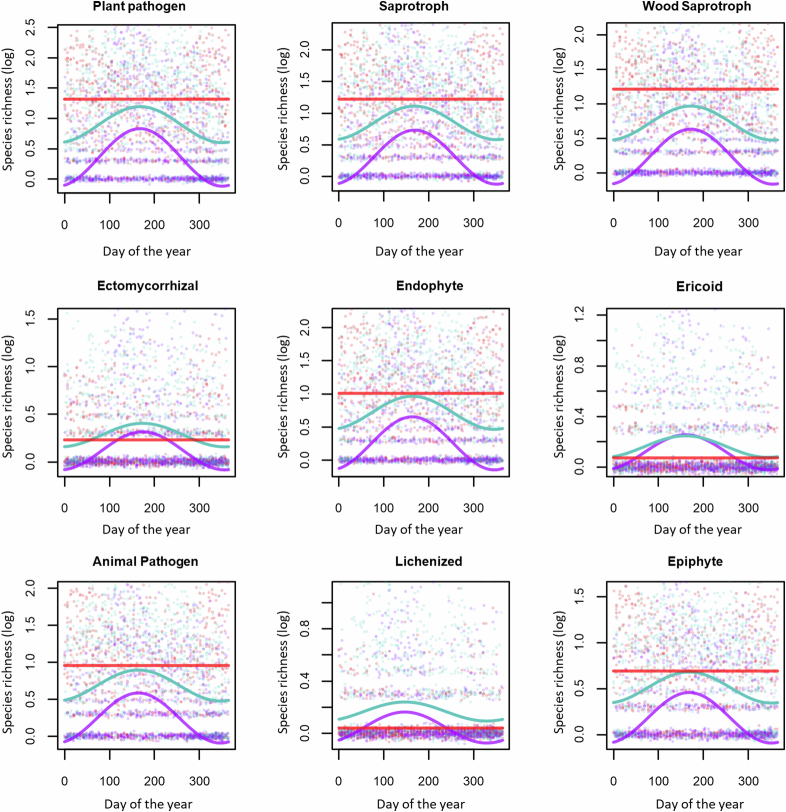
Seasonal variation in airborne fungal diversity of different trophic guilds, with data contaminated with sequences counts observed in negative controls. The lines in the panels show the predictions of the best-supported linear mixed models (see Methods) for tropical-subtropical (red), temperate (cyan), and polar-continental (purple) climatic zones. Note that the predictions are shown for the Northern Hemisphere, whereas for the Southern Hemisphere the seasonal patterns would be mirror images. The response variable in each panel is the species richness of the trophic guild shown in the panel’s titles. The dots in that show the raw data have been slightly jiggered to reveal overlap. For comparison with the original data, see Extended Data Fig. 5. The results are shown for one of the ten replicates of the contaminated datasets.

**Extended Data Fig. 6:**
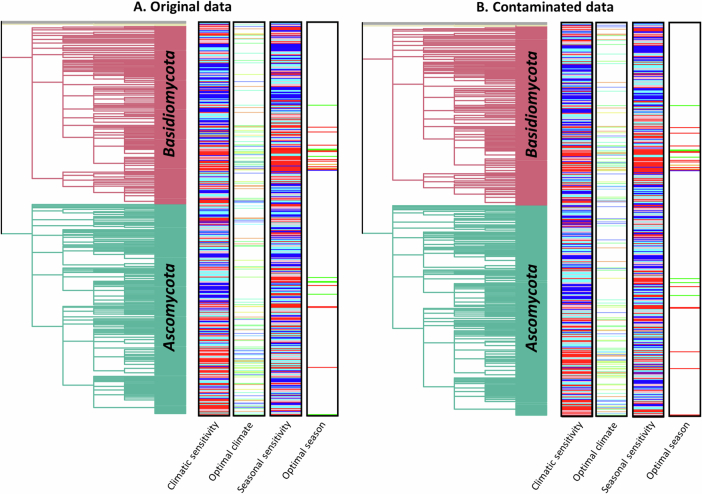
Comparison of HMSC analyses with original data (A; replicated from Fig. 4a of the main document) and data contaminated with sequences counts observed in negative controls (B). The results are shown for one of the ten replicates of the contaminated datasets.

We amplified the ITS2 region using PCR for 20 cycles with fusion primers ITS_S2F^60^, ITS3 and ITS4 (ref. ^61^) tailed with Illumina adaptors and sequenced them on Illumina MiSeq. In the MiSeq runs we included two sets of negative control samples, introduced at the DNA extraction step and the PCR step, respectively. Of the 99 negative control samples, 89% (88 samples) did not yield any reads of fungal origin. The remaining nine negative control samples included a few fungal reads (relative to the study samples) of relatively common OTUs, suggesting infrequent cross-contamination. To test the robustness of the results with respect to such cross-contamination, we repeated three of the main analyses (variation in overall species richness, variation in guild-specific species richness and joint-species distribution modelling) with data that we purposely contaminated with the observed level of cross-contamination. To do so we added to the OTU reads of each field sample the OTU reads of a randomly selected negative control sample. We replicated the cross-contamination simulation for ten independent replicates, with the results being almost identical to those obtained from the original data (Supplementary Information and Extended Data Figs. 4–6). To quantify the amount of fungal DNA we applied a spike-in approach and converted the ratio of non-spike versus spike sequences into semiquantitative estimates of DNA amount^35^. Demultiplexed paired-end reads were trimmed, denoised and chimera checked using Cutadapt v.4.2 (ref. ^62^), DADA2 v.1.18.0 (ref. ^63^) and VSEARCH v.2.22.1 (ref. ^64^). As a reference database we used Sanger sequences from the UNITE v.9 database^65^ supplemented with the synthetic spike sequences. Sequences representing non-spike ASVs^38^ were aligned between the ITS3 and ITS4 primer sites. Discarding of sequences that did not match the full length of the model, or with a bit score less than 50, resulted in a 65,912 ASV × 2,768 sample matrix of read abundance.

Due to the unsuitability of using ITS-based ASVs as proxies for species^66^, we developed a taxonomically guided clustering approach to form species-level OTUs. We performed a probabilistic taxonomic placement of ASVs with Protax-fungi^40^ with a 90% probability threshold. In addition, sequences whose best match to UNITE Sanger sequences was to a kingdom other than Fungi were annotated as potential non-fungi. We applied constrained clustering by first forming cluster cores by those ASVs that had been assigned to taxa by Protax-fungi. We then matched unassigned ASVs to the closest cluster core using optimized sequence similarity thresholds. Finally, remaining unclustered ASVs were clustered using de novo single-linkage clustering. These de novo clusters were assigned to placeholder taxonomic names of the form ‘pseudo{rank}_{number}’. The final result of this process was a read abundance matrix of 27,954 species-level OTUs × 2,768 samples, along with taxonomic annotations at each rank from phylum to species, including pseudotaxon placeholders.

The mean sequencing depth (total number of fungal and spike sequences) among samples was 86,845 sequences per sample. Based on rarefaction analyses presented in ref. ^3^ we discarded samples that did not contain at least 10,000 sequencing reads, representing 1.8% of samples. To avoid losing some OTUs detected in the most diverse samples, we controlled for variation in sequencing depth by statistical means rather than using rarefied values^67^.

### Weather and climate data

Weather variables were extracted from ERA5 hourly data on single levels dataset^42^ available at the Copernicus Climate Data Store (https://cds.climate.copernicus.eu/cdsapp#!/home). To download weather variables we used the R package ecmwfr^68^. We downloaded hourly data on (1) 2m_temperature—that is, instantaneous temperature (k) at 2 m height (henceforth termed temperature), (2) total_precipitation—that is, precipitation (m) accumulated over a 1 h period (henceforth termed precipitation), (3) 10m_v_component_of_wind—that is, horizontal speed (m s^−1^) of air moving towards the north at a height of 10 m and (4) 10m_u_component_of_wind—that is, horizontal speed (m s^−1^) of air moving towards the east at a height of 10 m. The latter two variables (wind to north *v* and wind to east *u*) were combined to compute wind speed by applying the formula $\sqrt{{v}^{2}+{u}^{2}}$. All four variables were downloaded for the latitude range from −80 to 80 degrees and longitude range from −180 to 180 degrees for the period 7 May 2018 to 2 February 2021, which extended well past our study period. We then averaged the hourly data to daily data and extracted data for the sampling locations of our study. We downloaded climatic data using the same tools but with the ‘sis biodiversity ERA5 global dataset’. As climatic variables we included the 40-year averages (1979–2018) of annual_mean_temperature (MAT), annual_precipitation (MAP), wind_speed and aridity (MAI).

### Extraction of spore size and trophic guild data

We extracted spore size and trophic guild data from the data assembled by Aguilar-Trigueros et al.^54^. Spore size data originate from species-level taxonomic descriptions in Mycobank^69^ (containing spore dimension data for over 36,000 species) and include, for every fungal species, the sizes of spores produced in both sexual and asexual cycles. The trophic guild data consist of a compilation of recordings of fungal functions across major databases (see ref. ^54^ for a detailed list of compiled databases).

Connecting spore volume data to molecularly identified species is not straightforward, because (1) some taxa were identified only to a higher taxonomic level than species and (2) the spore volume databases are not complete. For those OTUs identified to species level and for which a spore volume estimate was available we used the species-level estimate. When a species-level estimate was not available we used the genus-level estimate, computed as the average over the species belonging to the focal genus. When a genus-level estimate was not available we used the family-level estimate, computed as the average over the genera belonging to the focal family. If a family-level estimate was not available we considered the spore volume for the focal species as missing data. We computed the community-weighted mean (CWM) of log-transformed spore volume for each sample as the average log-transformed spore volume over the species present in the sample. When doing so we distinguished between spores produced during asexual (that is, asexual spores) and sexual cycles (that is, sexual spores), thus resulting in CWM sizes of both asexual and sexual spores. We note that this analysis is based on the molecular classifications of the ITS2 sequences rather than, for example, direct microscopy of the sampled spores, and hence we cannot distinguish whether the spores in the samples were asexual or sexual. Therefore, these variables should be interpreted as the mean size of the asexual or sexual spores of those species present in the sample.

When assigning the trophic guild data we included only the most common trophic guilds and grouped some of them (Extended Data Table 2). We first matched those OTUs identified to the species level and which matched a species in the database of Aguilar-Trigueros et al.^54^. In those cases for which an OTU was identified to only genus level, or species-level identification was not available in the database, we assigned from the database all trophic guild categories of the species belonging to the focal genus; likewise, when the OTU was identified only to the family level we assigned from the database all trophic guild categories of the species belonging to the focal family. As result, some OTUs were assigned to more than one trophic guild and hence the classifications should be considered as potential guilds to which the OTU may belong, often based on information borrowed from its relatives.

### Variation in community composition

We conducted multivariate analyses at the site, rather than at the sample, level. For each site we measured the abundance of each taxon by its prevalence—that is, the proportion of samples in which it was present. We then computed the site-to-site community distance matrix using either the Bray–Curtis dissimilarity index (using the vegdist function of the R package vegan^70^) or, alternatively, the unifrac distance (using the UniFrac function of the R package phyloseq^71^) that accounted for taxonomic relatedness among the taxa. As candidate environmental variables used to explain community dissimilarity we used MAT, MAP, MAI and mean annual wind speed, all averaged over the 40-year period from 1979 to 2018. The reason for including only a small number of site-specific variables in the analysis is that, whereas the study is global in scope, it includes only 47 sites. The data thus hold limited information on statistically disentangling the effects of many spatially varying covariates. Instead, the main strength of the study lies in its high temporal replication, which allowed us to identify effects of the spatiotemporal covariates such as seasonality.

We visualized the community distance matrices with non-metric multidimensional scaling (NMDS; using the metaMDS function of the R package vegan) and illustrated the effect of each candidate environmental variable on the ordination space (using the ordisurf function of the R package vegan). To partition the variation in community dissimilarity explained by spatial distance and by each candidate environmental variable, we used linear models in which community dissimilarity was explained by either geographic distance, environmental distance or both. We computed the proportions of variance explained by space alone, by environment alone and by shared effect following Whittaker^72^.

### Univariate analyses addressing how variations in DNA amount, species richness, spore size and trophic guild composition depend on climate, season and weather

We fitted a series of mixed linear models with the R package lme4 (ref. ^73^) for each of the following response variables: log(DNA amount), log(species richness + 1), CWM log(sexual spore size), CWM log(asexual spore size) and log(number of species classified to each trophic guild + 1). For analyses concerning spore size we included only samples that contained at least ten species, to reduce noise in the response variables. In addition to conducting the analyses for CWM computed for all species, we also repeated the spore size analyses with restriction for basidiomycetes only and for ascomycetes only. These additional analyses were motivated by the question of whether the results were consistent among these two major groups.

**Extended Data Fig. 7:**
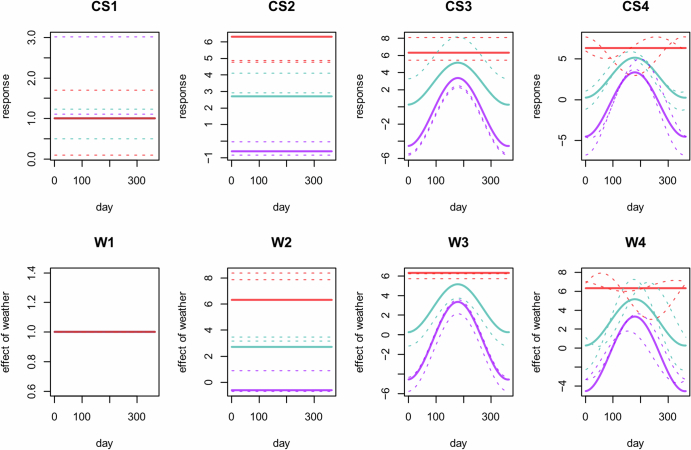
A conceptual illustration of different model variants. The upper panels depict the climatic models CS1 (Null; site-dependency as random effect), CS2 (Climate-dependency), CS3 (Climate-dependency and latitude-dependent seasonality) and CS4 (Climate-dependency and site-specific seasonality). The lower panels depict the weather models W1 (Constant weather effects), W2 (Weather effects depend on site), W3 (Weather effects depend on site and on latitude-dependent seasonality), and W4 (Weather effects depend on site and on site-dependent seasonality). Note that the weather models are not independent models as they are embedded within the climatic models. In each panel, the lines show hypothetical responses. The continuous lines depict the mean responses for the tropical-subtropical (red), temperate (cyan), and polar-continental (purple) climatic zones.

As described in greater detail below, we considered four models (CS1–CS4) of climatic and seasonal variation. In addition to the best-supported model of climatic and seasonal variation we considered four models (W1–W4) of weather variation, each of which further consisted of 64 variants according to which weather variables they included. We describe these model variants below and illustrate them conceptually in Supplementary Information and Extended Data Fig. 7. We performed model selection among these model variants with Akaike information criterion (AIC) and used the explanatory powers of the models to assess the proportion of total variation they explain.

#### Influence of climatic and seasonal variation

To evaluate the effects of climatic and seasonal variation we considered the following four nested models, described in order of increasing complexity.

1. Model CS1: null model. The null model does not include any ecological predictors as fixed effects but includes log(sequencing depth) for the species richness model. To account for the study design with multiple samples from the same locations, the null model includes the site as a random intercept.
2. Model CS2: climate dependence. In this model we assumed that the response variable varies systematically with the MAT of the site. Thus, we extended model CS1 by including a fixed effect of MAT and its square.
3. Model CS3: climate dependence and latitude-dependent seasonality. In this model we assumed that the response variable additionally shows seasonal variation that systematically depends on latitude. We thus extended model CS2 by including as fixed effects the interaction between latitude and seasonality. We modelled seasonality with the periodic functions $\sin \left(2\frac{{\rm{\pi }}d}{365}\right)$ and $\cos \left(2\frac{{\rm{\pi }}d}{365}\right)$, where *d* is the Julian day of the year. Because latitude is positive for the Northern and negative for the Southern Hemisphere, we note that the interaction between seasonality and latitude assumes opposite patterns of seasonality in the two hemispheres. It is thus appropriate to account for the 6 month difference in seasonality between the two hemispheres.
4. Model CS4: climate dependence and site-specific seasonality. Model CS4 extends model CS3 by including the random effect of the site not only in the intercept, but also as random slopes related to seasonality. This model thus assumes that each site may show a deviation from the systematic latitude-dependent variation in seasonality, generated by some site-specific effects not included in the model.

#### Influence of weather variation

The aim of these analyses was to assess how the prevailing weather conditions influence the four response variables. As weather-related covariates we used temperature, precipitation and wind speed and added these covariates as additional predictors to CS4, the most complex climatic model. Because weather variables (especially temperature) follow seasonal patterns that depend on latitude, using them as such would confound their effects with those of the climatic and seasonal predictors. For this reason we included the covariates as the difference between the actual values and those expected based on latitude and season; henceforth we term these temperature, precipitation and wind-speed anomalies. We calculated these anomalies as the differences between the daily observed values and the predictions of site-specific seasonality models (that is, model CS4) fitted to each weather covariate. For example, the temperature anomaly for a given day and site describes how much warmer (positive anomaly) or colder (negative anomaly) that site was compared with what would be expected for that site in that season. Furthermore, we note that the weather covariates may influence variation in fungal communities through either their effect on detection (for example, prevailing wind conditions during sampling) or their influence on production of fruiting bodies and sporulation (for example, temperature and humidity conditions over the past week). Because the timescales at which climatic conditions influence spore production are generally unknown and can vary among species, we computed the weather predictors in three alternative ways, averaging them over a period of either 1 day, 1 week or 1 month before sampling. We considered the full set of candidate models in which each weather covariate was either excluded or included at the time scale of day, week or month. Because there are three weather covariates and each of them has four options the number of candidate models is 64, encompassing the null model in which no weather covariates were included. In regard to how we assumed that weather would influence the response variables we considered the following four nested models, each of which included as baseline the best-supported model of climate and seasonality.

1. Model W1: constant weather effects. Model W1 includes in the fixed effects the main effects of weather covariates.
2. Model W2: weather effects depend on the site. Model W2 extends model W1 by also including in the fixed effects the interactions between climatic variables (MAT and its square) and weather covariates, as well as in the random effects the interactions between site and weather covariates, thus allowing temperature anomaly to have a site-specific effect that potentially varies systematically with climate.
3. Model W3: weather effects depend on both the site and latitude-dependent seasonality. Model W3 extends model W2 by also featuring inclusion in the fixed effects the interactions between latitude-dependent seasonality (the interaction between latitude and periodic functions of the day of the year) and weather covariates, thus allowing, for example, temperature anomaly to have a positive effect in spring but negative effect in autumn.
4. Model W4: weather effects depend on both the site and site−dependent seasonality. Model W4 extends model W3 by including in the random effects the effect of the site, and the slopes related to interaction between seasonality and the weather covariates. This model thus assumes that the effects of the weather covariates show site-specific variation in both their mean effect and seasonality.

### Seasonality in community composition

To characterize how seasonality in community composition is dependent on climate we computed for each site an index of seasonality in community composition and then fitted a linear model in which we regressed this index against the MAT of the site. To describe seasonality in community composition we examined how much more similar pairs of samples were in terms of their community composition if they were sampled from the same season compared with whether they were sampled from different seasons. We considered a pair of samples as belonging to the same season if they were taken at most 1 month apart, whereas we considered them as belonging to a different season if they were taken 3 months (plus or minus half a month) from each other. As a measure of community similarity we used the Jaccard similarity index, which we averaged over those pairs of samples that contained at least five species. We then used an index of seasonality in community composition calculated as the average Jaccard similarity index for pairs of samples that were taken in the same season, minus the average Jaccard similarity index for pairs of samples taken in a different season. We accounted for the Jaccard similarity index for pairs of samples that were taken in the same season to control for possible variation in the baseline turnover and thus to extract the sole effect of seasonality.

### Joint-species distribution modelling of phylogenetic signal in climatic and seasonal variation

To examine for phylogenetic signals in climatic and seasonal variation we analysed the data with HMSC^47,74^. HMSC is a joint-species distribution model^75^ that includes a hierarchical layer modelling how species’ environmental covariates relate to their traits and/or phylogenetic relationships^76^. We restricted these analyses to the 485 species that occurred in the data at least 50 times, and therefore had sufficient data to estimate climatic and seasonal responses. As the response variable we used the presence/absence of species at the level of the sample, which we modelled through the Bernoulli distribution and probit-link function. To measure climatic responses we included as fixed effects the second-order polynomial of the MAT of the site. To measure seasonal responses we also included as fixed effects the interaction between latitude and seasonality that we modelled with the periodic functions sin(2π*d*/365) and cos(2π*d*/365), where *d* is the Julian day of the year. To control for variation in sequencing depth (that is, the number of sequences obtained for each sample) we also included the log-transformed sequencing depth as fixed effect. To control for repeated samples from the same sites we included the site as a random effect. To examine how species’ responses to the predictors related to their phylogenetic relationships we included in the HMSC model a taxonomic tree in which we assumed equal branch lengths at the levels of phylum, class, order, family, genus and species.

We fitted the model with the R package HMSC^77^ assuming the default prior distributions^47^. We sampled the posterior distribution with four Markov chain Monte Carlo chains, each of which was run for 37,500 iterations of which the first 12,500 were removed as burn-in. The chains were thinned by 100 to yield 250 posterior samples per chain and so 1,000 posterior samples in total. We examined the convergence of Markov chain Monte Carlo by the potential scale-reduction factors^78^ of the model parameters. We examined the explanatory power of the model through species-specific area under the curve^79^ and Tjur’s *R*^2^ metric^80^ values, which provide complementary insights of predictive performance^81^.

To quantify the phylogenetic signals of climatic and seasonal variation we extracted four output variables for each species from the fitted HMSC models: climatic sensitivity, optimal climate, seasonal sensitivity and optimal season. We measured climatic sensitivity by the proportion of variance explained by the second-order polynomial of the MAT of the site. Similarly we measured seasonal sensitivity by the proportion of variance explained by the periodic functions sin(2π*d*/365) and cos(2π*d*/365). We multiplied the proportions of variance explained by the predictors out of the explained variation by the proportion of variation explained by the model, the latter measured by species-specific Tjur’s *R*^2^ values. We measured optimal climate as the MAT at which the second-order polynomial of the MAT was maximized, truncated to values within the observed range of MATs. Because it is meaningful to estimate the optimal climate only for species that show climatic variation, we included in the analyses of optimal climate only those species for which climatic sensitivity was at least 5%. Similarly we measured optimal season by the day of the year on which the estimated linear combination of the periodic functions sin(2π*d*/365) and cos(2π*d*/365) peaked, and included in the analyses of optimal season only those species for which seasonal sensitivity was at least 5%. We then fitted phylogenetic regression models for each of these four response variables and fitted the models with the R package nlme^73^ using the gls function, no covariates and the corPagel correlation structure. We quantified the strength of the phylogenetic signal by the estimated λ parameter and estimated its statistical significance by the *P* value of the comparison (performed by the analysis of variance function) between models that included versus did not include the corPagel correlation structure.

### Reporting summary

Further information on research design is available in the Nature Portfolio Reporting Summary linked to this article.

## Online content

Any methods, additional references, Nature Portfolio reporting summaries, source data, extended data, supplementary information, acknowledgements, peer review information; details of author contributions and competing interests; and statements of data and code availability are available at 10.1038/s41586-024-07658-9.

### Supplementary information

Supplementary Information The Supplementary Information contains text (three items), Supplementary Tables 1–7 and additional details on methods and results.

Reporting Summary

Peer Review File

## Figures and Tables

**Extended Data Table 1:**
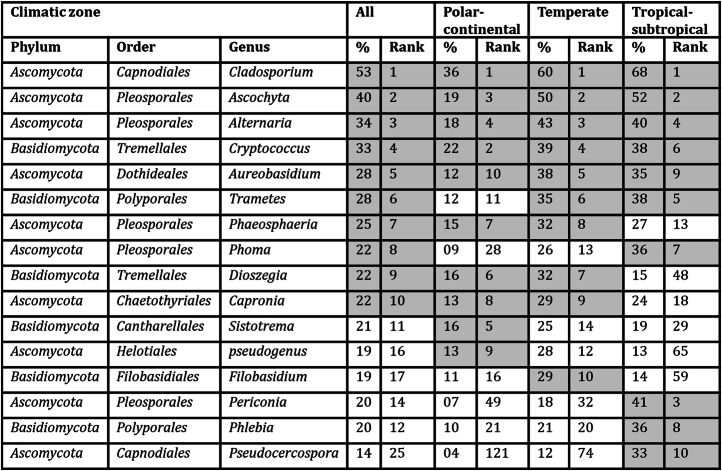
Most common genera found in the GSSP data

The table shows the prevalence (%) of each genus, computed as the fraction of samples in which it was detected, as well as the ranking of the genus in terms of its prevalence. The prevalences and rankings are shown for all samples, as well as separately for the samples from each of the three climatic zones. Genera that rank in the top ten are highlighted, and only genera that ranked in the top ten in at least one of the climatic zones are included.

**Extended Data Table 2:**
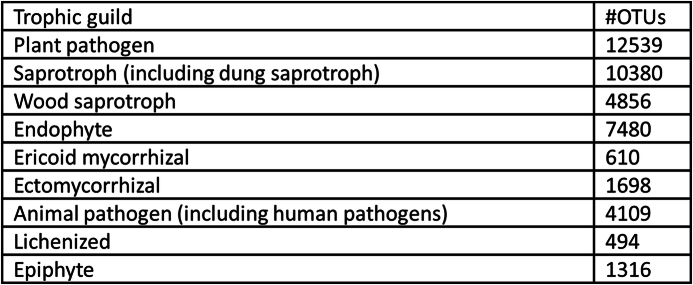
The numbers of OTUs classified into trophic guilds used in this study

Note that each OTU may be classified to more than one trophic guild and hence the sum of #OTUs over the trophic guilds exceeds the total number of OTUs detected in our study.

## Data Availability

All data used in this paper are available at Zenodo (10.5281/zenodo.10444737)^59^. GSSP data were downloaded from Ovaskainen et al.^3^. Climatic data were downloaded from the Copernicus Climate Change Service Climate Data Store^42^ (‘ERA5 hourly data on single levels dataset’ and ‘sis biodiversity era5 global dataset’). We extracted spore size and trophic guild data from data assembled by Aguilar-Trigueros et al.^54^. Spore size data originate from species-level taxonomic descriptions in Mycobank^69^.

## Code availability

The R pipeline that can be used to reproduce the results of this paper is available at Zenodo (10.5281/zenodo.10444737)^59^. All analyses were conducted in R v.4.3.1 (ref. ^82^) with the packages ade4 1.7-22, adespatial 0.3-23, ape 5.7-1, ecmwfr 1.5.0, geosphere 1.5-18, Hmsc 3.0-14, jsonify 1.2.2, kgc 1.0.0.2, lme4 1.1-35.1, lubridate 1.9.3, maps 3.4.2, MASS 7.3-60, ncdf4 1.22, nlme 3.1-162, phyloseq 1.46.0, phytools 2.1-1, raster 3.6-26, rgdal 1.6-7, scales 1.3.0, sjstats 0.18.2, tidyverse 2.0.0, vegan 2.6.4, VennDiagram 1.7.3, vioplot 0.4.0 and wordcloud 2.6.
