# Supplementary material for: Airborne DNA reveals predictable spatial and seasonal dynamics of fungi
Source: Nature. 2024 Jul 10;631(8022):835–42. doi: 10.1038/s41586-024-07658-9 (PMC11269176; doi:10.1038/s41586-024-07658-9)
Supplement: Supplementary file 1 — The Supplementary Information contains text (three items), Supplementary Tables 1–7 and additional details on methods and results. [file 41586_2024_7658_MOESM1_ESM.pdf]

nature portfolio

https://doi.org/10.1038/s41586-024-07658-9

Supplementary information

Airborne DNA reveals predictable spatial and seasonal dynamics of fungi

In the format provided by the authors and unedited

Supplementary text

Robustness of ordination analyses with respect to the distance measure

The Bray-Curtis dissimilarity and Unifrac distance yielded highly consistent results on site-specific ordinations (Extended Data Figure 1). With both distance measures, the mean annual air temperature (MAT) of the site explained the major proportion of deviance in the ordination space (78%-80%), the mean annual precipitation (MAP) of the site clearly smaller proportion of deviance (41%-44%), and the mean annual wind speed (MAW) of the site yet clearly smaller proportion of deviance (23%-27%).

Additional information on the models of climatic and seasonal variation

Model conceptualization. As described in the main text, we considered four models (Models CS1 through CS4) of climatic and seasonal variation. Additionally, we used CS4 as a baseline for four models (Models W1 through W4) of weather variation, each of which further consisted of 64 variants according to which weather variables they included. Extended Data Figure 7 illustrates conceptually Models CS1-CS4 and Models W1-W4.

Additional results for the climatic and seasonal models. Tables S1 and S2 summarize the statistical results of the climatic models based on an AIC comparison and on an R^2-comparison, respectively. Extended Data Figures 2 and 3 illustrate the community weighted mean spore sizes and richness of trophic guilds, respectively. Table S3 summarizes the results from the fitted weather models.

Robustness of the results with respect to cross-contamination

A common practice to evaluate the robustness of the results with respect to cross-contamination is to filter out potential false positives, defined e.g. as OTUs that have less sequences than in some of the negative controls, as for such cases the apparently observed species may actually represent cross-contamination[1]. However, in our main interest is not whether contamination may have introduced some particular species to some particular samples, but whether the global spatio-temporal patterns revealed by the statistical analyses are robust to cross-contamination. To do so, we followed the next approach.

We repeated three of the main analyses (variation in overall species richness, variation in guild specific species richness, and joint species distribution modelling) with data to which we purposely added simulated contamination. If the conclusions derived from the analyses conducted with contaminated data differed with those derived from the analyses conducted with original data, we considered the results not to be robust with contamination. To simulate the observed level of cross-contamination, we added to the OTU reads of each field sample the OTU reads of a randomly selected negative control sample. To evaluate the generality of the results, we simulated ten independent replicates of contaminated datasets, and then repeated the statistical analyses for each of them. Due to the computational intensiveness of the joint species distribution analyses, we fitted them for the ten contaminated datasets with thin=10, whereas thin=100 was applied for the main analyses applied to the original data. We note that this approach was conservative, as any effect of non-satisfactory MCMC converge (which is more likely with thin=10 than with thin=100) would increase the difference between results based on contaminated and original data.

The noise generated by simulated contamination was visible in the ΔAIC values (Table S5) and to a lesser amount in the R^2 values (Tables S6), but the qualitative results were highly robust. In case of both original

and contaminated data, the best model in sense of AIC was the weather model with positive effect of temperature and wind of the sampling day (Table S7). The predictions of seasonal and spatial variation in species richness were essentially identical for the original data and the contaminated data (Extended Data Figure 4). As was the case of the original data, all ten replicates of the contaminated datasets predicted species richness to be highest in the tropical zone.

As with overall species richness, also the trophic guild specific analyses of species richness showed that the results were highly robust with respect to the level of cross-contamination detected in the blank samples (Table S5, Table S6, Table S7). The predicted patterns of seasonal and spatial variation are visually almost identical between the original data (Extended Data Figure 3) and the contaminated data (Extended Data Figure 5). For all guilds, the climatic zone in which the predicted species richness was the highest was consistent between the original data and the contaminated data, with no exceptions among the ten replicates of contaminated data. Where we found minor differences, were in the effects of the weather variables (Table S7). Out of the 30 cases examined (three candidate weather variables for 10 response variables), 27 cases yielded identical results between all ten replicates of the contaminated and original data. For the remaining three cases, the ten replicates of the contaminated datasets differed in their results: it remained uncertain whether plant pathogens and endophytes responded negatively to precipitation, and whether ectomycorrhizal species responded positively to wind. However, in all of these three cases, the majority of the ten replicates of the contaminated data yielded the same result as the original data.

In the joint species distribution model HMSC, the model with the original data included 485 species, whereas the models with the contaminated data included on average 489 (min=488, max=490) species. Thus, the random addition of the OTU reads present in the negative controls increased the number of occurrences for four to five additional species over the selected threshold of 50 occurrences. The explanatory power (averaged over the species) for the original model was AUC=0.90 and Tjur's R^2=0.16, whereas for the model with contaminated data it was AUC=0.89 (min 0.893, max=0.894) and Tjur's R^2=0.16 (min=0.157, max=0.158). With the original data the phylogenetic signal in climatic sensitivity was estimated to be Pagel's lambda=0.28 (p=4E-12), whereas among the contaminated datasets the mean estimate of Pagel's lambda was 0.24 (min=0.20, max=0.28), with p<1E-7 for all datasets. With the original data the phylogenetic signal in optimal climate was estimated to be Pagel's lambda=-0.01 (p=0.81), whereas among the contaminated datasets the mean estimate of Pagel's lambda was 0.04 (min=-0.08, max=0.31), with p>0.05 for all but two datasets. With the original data phylogenetic signal in seasonal sensitivity was estimated to be Pagel's lambda=0.39 (p=2E-16), whereas among the contaminated datasets the mean estimate of Pagel's lambda was 0.38 (min=0.35, max=0.41), with p<1E-10 for all datasets. With the original data the phylogenetic signal in optimal season was estimated to be Pagel's lambda=-0.04 (p=0.80), whereas among the contaminated datasets the mean estimate of Pagel's lambda was -0.06 (min=-0.11, max=-0.02), with p>0.05 for all but two datasets. Thus, we conclude that the findings of moderate phylogenetic signal for climatic sensitivity, high phylogenetic signal for seasonal sensitivity, and no phylogenetic signal for optimal season are robust with respect to potential cross-contamination. We note that for optimal season two of the contaminated datasets led to a statistically significant result, but as for these datasets the estimate of Pagel's lambda was negative, also these cases support no phylogenetic signal in optimal season. The finding of no phylogenetic signal for optimal climate is also likely to be robust as eight out of the ten contaminated datasets did not yield statistically significant results, and one out of the two statistically significant cases led to a negative estimate. However, as one out of the ten the contaminated datasets led to statistically significant positive estimate, we cannot fully exclude the possibility that our finding of no phylogenetic signal in optimal climate may have been influenced by cross-contamination. Extended Data Figure 6 exemplifies, for one of the contaminated datasets, the very close correspondence of the result obtained for original and contaminated datasets.

References

1. Hänfling, B., Lawson Handley, L., Read, D. S., Hahn, C., Li, J., Nichols, P., Blackman, R. C., Oliver, A., & Winfield, I. J. (2016). Environmental DNA metabarcoding of lake fish communities reflects long-term data from established survey methods. Molecular Ecology, 25(13), 3101–3119. https://doi.org/10.1111/mec.13660

2. Lüdecke, D. (2018). sjstats: Statistical Functions for Regression Models. (0.17.2) [Computer software]. https://doi.org/10.5281/ZENODO.1284472

Supplementary Tables

Table S1. AIC-based comparison between univariate models fitted to DNA amount, species richness, and spore sizes. The AIC-values are shown for the climatic models CS1-CS4 as well as for the best supported weather model variant of the model CS4 (W.best). The values are shown relative to the AIC value of model CS1.

Model/Response	CS1	CS2	CS3	CS4	W.best
Overall diversity					
DNA amount	0	0.68	-257.71	-456.19	-486.24
Species richness	0	4.08	-233.05	-429.25	-479.34
Trophic guild-specific species richness					
Plant pathogen	0	6.7	-261.69	-480.11	-519.61
Saprotroph	0	8.54	-226.55	-421.1	-462.17
Wood saprotroph	0	1.18	-253.98	-454	-497.71
Endophyte	0	10.66	-243.5	-468.36	-513.4
Ectomycorrhizal	0	24.73	-100.48	-183.9	-207.15
Animal pathogen	0	11.22	-199.21	-434.32	-473.32
Lichenized	0	17.99	-117.68	-243.47	-302.51
Epiphyte	0	14.27	-184.79	-428.58	-464.2
Ericoid mycorrhizal	0	21.77	-131.05	-254.04	-272.73
Spore size					
Asexual	0	7.84	-167.17	-202.03	-205.58
Sexual	0	26.94	18.72	-99.44	-105.91
Asexual basidiomycetes	0	25.78	-59.69	-68.13	-70.54
Sexual basidiomycetes	0	21.29	26.34	-29.81	-29.81
Asexual ascomycetes	0	18.36	-71.06	-85.04	-87.07
Sexual ascomycetes	0	10.93	-34.9	-55.56	-55.56

Table S2. R^2-based comparison between univariate models fitted to DNA amount, species richness, and spore sizes. The R^2-values are shown for the climatic models CS1-CS4 as well as for the best supported weather model variant of the model CS4 (W.best) The R^2-values were computed with the r2-function of the R-package sjstats[2].

Model/Response	CS1	CS2	CS3	CS4	W.best
Overall diversity					
DNA amount	0.29	0.29	0.37	0.38	0.39
Species richness	0.3	0.29	0.37	0.39	0.4
Trophic guild-specific species richness					
Plant pathogen	0.3	0.29	0.37	0.4	0.41
Saprotroph	0.3	0.3	0.37	0.39	0.41
Wood saprotroph	0.33	0.32	0.39	0.43	0.44
Endophyte	0.31	0.31	0.38	0.41	0.42
Ectomycorrhizal	0.16	0.16	0.21	0.22	0.24
Animal pathogen	0.32	0.31	0.38	0.4	0.41
Lichenized	0.19	0.2	0.25	0.25	0.27
Epiphyte	0.31	0.31	0.37	0.39	0.4
Ericoid mycorrhizal	0.14	0.15	0,21	0.21	NA
Spore size					
Asexual	0.33	0.33	0.4	0.42	0.42
Sexual	0.49	0.5	0.52	0.55	0.56
Asexual basidiomycetes	0.19	0.21	0.26	0.26	0.27
Sexual basidiomycetes	0.39	0.39	0.39	0.4	0.4
Asexual ascomycetes	0.28	0.28	0.33	0.34	0.35
Sexual ascomycetes	0.27	0.25	0.28	0.28	0.28

Table S3. Results from the fitted weather models. The cells for the rows *Temperature*, *Precipitation,* and *Wind* indicate the effect sign ("+" for positive and "-" for negative) and temporal scale at which the weather covariate was included in the best supported weather model, with an empty cell indicating that the weather covariate was excluded from the model.

Variable/Response	Tempe-rature	Precipi-tation	Wind	Best weather model
Overall diversity				
DNA amount	1	0	1	W1_temp:day_prec:no_wind:day
Species richness	1	0	1	W1_temp:day_prec:no_wind:day
Trophic guild-specific species richness				
Plant pathogen	1	0	1	W1_temp:day_prec:no_wind:day
Saprotroph	1	0	1	W1_temp:day_prec:no_wind:day
Wood saprotroph	1	0	0	W1_temp:day_prec:no_wind:no
Endophyte	1	-1	1	W1_temp:day_prec:week_wind:day
Ectomycorrhizal	1	0	1	W1_temp:day_prec:no_wind:day
Animal pathogen	1	0	1	W1_temp:day_prec:no_wind:day
Lichenized	1	-1	1	W1_temp:day_prec:day_wind:day
Epiphyte	1	0	1	W1_temp:day_prec:no_wind:day
Ericoid mycorrhizal	1	0	0	W2_temp:day_prec:no_wind:no
Spore size				
Asexual	0	0	-1	W1_temp:no_prec:no_wind:day
Sexual	0	-1	1	W1_temp:no_prec:month_wind:day
Asexual basidiomycetes	1	0	0	W1_temp:day_prec:no_wind:no
Sexual basidiomycetes	0	0	0	W1_temp:no_prec:no_wind:no
Asexual ascomycetes	0	0	-1	W1_temp:no_prec:no_wind:day
Sexual ascomycetes	0	0	0	W1_temp:no_prec:no_wind:no

Table S4. Taxa that occur in the GSSP data at least 50 times and were thus included in the Hmsc analyses. The taxa are listed in the same order as they are shown in Figure 4 of the main document.

phylum	order	genus	species
pseudophylum_0014	pseudoorder_0047	pseudogenus_00200	pseudospecies_00541
pseudophylum_0007	pseudoorder_0025	pseudogenus_00103	pseudospecies_00304
pseudophylum_0012	pseudoorder_0043	pseudogenus_00182	pseudospecies_00497
Zygomycota	Mucorales	Choanephora_20117	Choanephora_cucurbitarum_170049
Zygomycota	Mucorales	Rhizopus_20487	Rhizopus_microsporus_177331
Zygomycota	Mucorales	Rhizopus_20487	Rhizopus_arrhizus_167790
Basidiomycota	Ustilaginales	Ustilago_16391	Ustilago_nuda_199384
Basidiomycota	Ustilaginales	Ustilago_16391	pseudospecies_00425
Basidiomycota	Ustilaginales	Sporisorium_16327	pseudospecies_00194
Basidiomycota	Urocystidales	Urocystis_16381	pseudospecies_00238
Basidiomycota	Agaricostilbales	Kondoa_25451	Kondoa_aeria_464811
Basidiomycota	Agaricostilbales	Kondoa_25451	pseudospecies_00081
Basidiomycota	Pucciniales	Puccinia_16284	pseudospecies_00563
Basidiomycota	Pucciniales	Puccinia_16284	pseudospecies_00206
Basidiomycota	Pucciniales	Puccinia_16284	pseudospecies_00065
Basidiomycota	Pucciniales	Puccinia_16284	pseudospecies_00409
Basidiomycota	Pucciniales	Pucciniastrum_16285	Pucciniastrum_areolatum_232153
Basidiomycota	Wallemiales	Wallemia_10428	Wallemia_sebi_325537
Basidiomycota	Wallemiales	Wallemia_10428	Wallemia_muriae_344766
Basidiomycota	Exobasidiales	Exobasidium_17576	pseudospecies_00600
Basidiomycota	Golubeviales	Golubevia_812694	Golubevia_pallescens_812695
Basidiomycota	Golubeviales	Golubevia_812694	pseudospecies_00228
Basidiomycota	Microstromatales	Microstroma_11129	Microstroma_album_165301
Basidiomycota	Microstromatales	Microstroma_11129	Microstroma_phylloplanum_812713
Basidiomycota	dummy_order_Tilletiopsis	Tilletiopsis_10237	Tilletiopsis_washingtonensis_306864
Basidiomycota	Entylomatales	Entyloma_16122	pseudospecies_00029
Basidiomycota	Entylomatales	Entyloma_16122	pseudospecies_00058
Basidiomycota	Malasseziales	Malassezia_8831	Malassezia_restricta_437921
Basidiomycota	Cystobasidiales	Cystobasidium_17446	Cystobasidium_minuta_809340
Basidiomycota	Cystobasidiales	Cystobasidium_17446	Cystobasidium_pinicola_809344
Basidiomycota	Cystobasidiales	Cystobasidium_17446	Cystobasidium_slooffiae_809341
Basidiomycota	Cystobasidiales	Cystobasidium_17446	Cystobasidium_laryngis_809345
Basidiomycota	Cystobasidiales	Cystobasidium_17446	pseudospecies_00204
Basidiomycota	Erythrobasidiales	Erythrobasidium_11244	Erythrobasidium_hasegawianum_354939
Basidiomycota	dummy_order_Symmetrosporaceae	Symmetrospora_813117	Symmetrospora_gracilis_813120
Basidiomycota	dummy_order_Symmetrosporaceae	Symmetrospora_813117	Symmetrospora_coprosmae_813118
Basidiomycota	dummy_order_Buckleyzymaceae	Buckleyzyma_813127	Buckleyzyma_aurantiaca_813128
Basidiomycota	Leucosporidiales	Leucosporidium_16187	pseudospecies_00423
Basidiomycota	Leucosporidiales	Leucosporidium_16187	pseudospecies_00153
Basidiomycota	Leucosporidiales	Leucosporidium_16187	Leucosporidium_golubevii_372915
Basidiomycota	Leucosporidiales	Leucosporidium_16187	pseudospecies_00250
Basidiomycota	dummy_order_Curvibasidium	Curvibasidium_28863	pseudospecies_00242
Basidiomycota	Sporidiobolales	Rhodosporidiobolus_813359	Rhodosporidiobolus_odoratus_813377
Basidiomycota	Sporidiobolales	Rhodosporidiobolus_813359	Rhodosporidiobolus_colostri_813375
Basidiomycota	Sporidiobolales	Rhodotorula_9741	Rhodotorula_mucilaginosa_271749
Basidiomycota	Sporidiobolales	Rhodotorula_9741	pseudospecies_00114
Basidiomycota	Sporidiobolales	Sporobolomyces_10025	pseudospecies_00022
Basidiomycota	Sporidiobolales	Sporobolomyces_10025	Sporobolomyces_roseus_306337
Basidiomycota	pseudoorder_0029	pseudogenus_00130	pseudospecies_00378
Basidiomycota	pseudoorder_0050	pseudogenus_00218	pseudospecies_00587
Basidiomycota	pseudoorder_0013	pseudogenus_00044	pseudospecies_00140
Basidiomycota	Corticiales	pseudogenus_00106	pseudospecies_00315
Basidiomycota	Corticiales	Vuilleminia_18743	pseudospecies_00229
Basidiomycota	Auriculariales	Elmerina_17548	Elmerina_caryae_354655

Basidiomycota	Auriculariales	Exidia_17572	Exidia_glandulosa_207527
Basidiomycota	dummy_order_Peniophorella	Peniophorella_18202	Peniophorella_pubera_212717
Basidiomycota	dummy_order_Peniophorella	Peniophorella_18202	Peniophorella_praetermissa_510106
Basidiomycota	pseudoorder_0112	pseudogenus_00414	pseudospecies_01093
Basidiomycota	Atheliales	pseudogenus_00055	pseudospecies_00176
Basidiomycota	Atheliales	Athelia_17118	Athelia_decipiens_293539
Basidiomycota	Atheliales	pseudogenus_00029	pseudospecies_00109
Basidiomycota	pseudoorder_0007	pseudogenus_00025	pseudospecies_00102
Basidiomycota	pseudoorder_0008	pseudogenus_00032	pseudospecies_00119
Basidiomycota	Hymenochaetales	Fuscoporia_17626	Fuscoporia_ferruginosa_122487
Basidiomycota	Hymenochaetales	Phellinus_18246	Phellinus_gilvus_120542
Basidiomycota	Hymenochaetales	Pseudochaete_28587	Pseudochaete_tabacina_484934
Basidiomycota	Hymenochaetales	Trichaptum_18675	Trichaptum_abietinum_324865
Basidiomycota	Hymenochaetales	Sidera_516944	Sidera_vulgaris_516948
Basidiomycota	Hymenochaetales	Basidioradulum_17150	Basidioradulum_crustosum_521705
Basidiomycota	Hymenochaetales	Schizopora_18514	Schizopora_paradoxa_338860
Basidiomycota	Hymenochaetales	Xylodon_18771	Xylodon_asperus_509767
Basidiomycota	Hymenochaetales	Xylodon_18771	Xylodon_nespori_509784
Basidiomycota	Hymenochaetales	Xylodon_18771	Xylodon_sambuci_563010
Basidiomycota	Hymenochaetales	Kneiffiella_17877	Kneiffiella_flavipora_537003
Basidiomycota	Hymenochaetales	Hyphodontia_17826	pseudospecies_00331
Basidiomycota	Hymenochaetales	Hyphodontia_17826	Hyphodontia_radula_436325
Basidiomycota	Hymenochaetales	Hyphodontia_17826	Hyphodontia_pallidula_298796
Basidiomycota	Trechisporales	Sistotremastrum_18553	pseudospecies_00190
Basidiomycota	Trechisporales	Sistotremastrum_18553	Sistotremastrum_niveocremeum_305982
Basidiomycota	Trechisporales	Sistotremastrum_18553	Sistotremastrum_guttuliferum_800173
Basidiomycota	Agaricales	Bovista_19036	pseudospecies_01551
Basidiomycota	Agaricales	Bovista_19036	pseudospecies_00094
Basidiomycota	Agaricales	Bovista_19036	pseudospecies_00085
Basidiomycota	Agaricales	pseudogenus_00056	pseudospecies_00177
Basidiomycota	Agaricales	Chondrostereum_17295	Chondrostereum_purpureum_294820
Basidiomycota	Agaricales	Mycena_18084	pseudospecies_00299
Basidiomycota	Agaricales	Panellus_18178	pseudospecies_00280
Basidiomycota	Agaricales	Panellus_18178	Panellus_stipticus_355858
Basidiomycota	Agaricales	Panellus_18178	Panellus_serotinus_433470
Basidiomycota	Agaricales	Plicaturopsis_18311	Plicaturopsis_crispa_337267
Basidiomycota	Agaricales	Hypholoma_17828	Hypholoma_capnoides_159037
Basidiomycota	Agaricales	Hypholoma_17828	Hypholoma_lateritium_455825
Basidiomycota	Agaricales	Hypholoma_17828	Hypholoma_fasciculare_152334
Basidiomycota	Agaricales	Coprinopsis_17366	pseudospecies_00169
Basidiomycota	Agaricales	Coprinopsis_17366	Coprinopsis_cinerea_474379
Basidiomycota	Agaricales	Coprinopsis_17366	Coprinopsis_atramentaria_474167
Basidiomycota	Agaricales	Psathyrella_18378	pseudospecies_00611
Basidiomycota	Agaricales	Coprinellus_17364	pseudospecies_00232
Basidiomycota	Agaricales	Coprinellus_17364	Coprinellus_disseminatus_107842
Basidiomycota	Agaricales	Coprinellus_17364	pseudospecies_00547
Basidiomycota	Agaricales	Coprinellus_17364	pseudospecies_00059
Basidiomycota	Agaricales	Cylindrobasidium_17430	Cylindrobasidium_evolvens_312444
Basidiomycota	Agaricales	Strobilurus_18610	pseudospecies_00046
Basidiomycota	Agaricales	Strobilurus_18610	pseudospecies_00028
Basidiomycota	Agaricales	Baeospora_17144	Baeospora_myosura_271938
Basidiomycota	Agaricales	Schizophyllum_18512	Schizophyllum_commune_208403
Basidiomycota	Russulales	Peniophora_18201	Peniophora_incarnata_198384
Basidiomycota	Russulales	Peniophora_18201	pseudospecies_00627
Basidiomycota	Russulales	Heterobasidion_17745	Heterobasidion_annosum_119859
Basidiomycota	Russulales	Heterobasidion_17745	pseudospecies_00030
Basidiomycota	Russulales	Stereum_18596	Stereum_sanguinolentum_205630
Basidiomycota	Russulales	Stereum_18596	Stereum_hirsutum_189826
Basidiomycota	Cantharellales	Rhizoctonia_9725	pseudospecies_00959

Basidiomycota	Cantharellales	Rhizoctonia_9725	pseudospecies_01817
Basidiomycota	Cantharellales	Ceratobasidium_17260	pseudospecies_01139
Basidiomycota	Cantharellales	Ceratobasidium_17260	pseudospecies_00713
Basidiomycota	Cantharellales	Thanatephorus_18639	Thanatephorus_cucumeris_306777
Basidiomycota	Cantharellales	Botryobasidium_17183	Botryobasidium_intertextum_111167
Basidiomycota	Cantharellales	Botryobasidium_17183	Botryobasidium_subcoronatum_254907
Basidiomycota	Cantharellales	Clavulina_17322	pseudospecies_00336
Basidiomycota	Cantharellales	Clavulina_17322	pseudospecies_00124
Basidiomycota	Cantharellales	Clavulina_17322	pseudospecies_00063
Basidiomycota	Cantharellales	Sistotrema_18551	pseudospecies_00266
Basidiomycota	Cantharellales	Sistotrema_18551	pseudospecies_00234
Basidiomycota	Cantharellales	Sistotrema_18551	Sistotrema_epiphyllum_809154
Basidiomycota	Cantharellales	Sistotrema_18551	pseudospecies_00147
Basidiomycota	Cantharellales	Sistotrema_18551	Sistotrema_sernanderi_305992
Basidiomycota	Cantharellales	Sistotrema_18551	Sistotrema_brinkmannii_305986
Basidiomycota	dummy_order_Resinicium	Resinicium_18453	Resinicium_friabile_442461
Basidiomycota	dummy_order_Resinicium	Resinicium_18453	Resinicium_bicolor_338261
Basidiomycota	Polyporales	pseudogenus_00081	pseudospecies_00244
Basidiomycota	Polyporales	Rigidoporus_18478	Rigidoporus_sanguinolentus_338623
Basidiomycota	Polyporales	pseudogenus_00054	pseudospecies_00172
Basidiomycota	Polyporales	Xenasmatella_18756	Xenasmatella_ardosiaca_474098
Basidiomycota	Polyporales	Xenasmatella_18756	Xenasmatella_borealis_517402
Basidiomycota	Polyporales	Ceriporia_17267	Ceriporia_viridans_356865
Basidiomycota	Polyporales	Ceriporiopsis_17268	Ceriporiopsis_subvermispora_105132
Basidiomycota	Polyporales	Ceriporiopsis_17268	Ceriporiopsis_gilvescens_327978
Basidiomycota	Polyporales	Phlebiopsis_25478	Phlebiopsis_gigantea_319885
Basidiomycota	Polyporales	Phanerochaete_18245	pseudospecies_00127
Basidiomycota	Polyporales	Ganoderma_17639	Ganoderma_adspersum_314302
Basidiomycota	Polyporales	Ganoderma_17639	pseudospecies_00034
Basidiomycota	Polyporales	Postia_18356	Postia_caesia_438906
Basidiomycota	Polyporales	Ischnoderma_17864	Ischnoderma_benzoinum_315943
Basidiomycota	Polyporales	Fomitopsis_17612	Fomitopsis_pinicola_101927
Basidiomycota	Polyporales	Antrodia_17083	Antrodia_serialis_326342
Basidiomycota	Polyporales	Antrodia_17083	Antrodia_xantha_308787
Basidiomycota	Polyporales	Fomitopsis_18291	Fomitopsis_betulina_812646
Basidiomycota	Polyporales	Microporus_18063	Microporus_xanthopus_456171
Basidiomycota	Polyporales	Cerioporus_17266	Cerioporus_mollis_812039
Basidiomycota	Polyporales	Lentinus_17925	pseudospecies_00189
Basidiomycota	Polyporales	Panus_18180	Panus_ciliatus_413663
Basidiomycota	Polyporales	Cerrena_17270	Cerrena_unicolor_356790
Basidiomycota	Polyporales	Cerrena_17270	pseudospecies_00170
Basidiomycota	Polyporales	Skeletocutis_18554	Skeletocutis_nivea_323593
Basidiomycota	Polyporales	Cinereomyces_17303	Cinereomyces_lindbladii_110544
Basidiomycota	Polyporales	Fomes_17608	Fomes_fomentarius_194860
Basidiomycota	Polyporales	Hexagonia_17755	pseudospecies_00044
Basidiomycota	Polyporales	Amyloporia_17070	Amyloporia_sinuosa_519381
Basidiomycota	Polyporales	Trametes_18663	pseudospecies_00184
Basidiomycota	Polyporales	Trametes_18663	pseudospecies_00181
Basidiomycota	Polyporales	Trametes_18663	Trametes_polyzona_561896
Basidiomycota	Polyporales	Trametes_18663	Trametes_ochracea_132931
Basidiomycota	Polyporales	Trametes_18663	pseudospecies_00488
Basidiomycota	Polyporales	Trametes_18663	pseudospecies_00158
Basidiomycota	Polyporales	Trametes_18663	Trametes_hirsuta_531523
Basidiomycota	Polyporales	Trametes_18663	Trametes_gibbosa_151431
Basidiomycota	Polyporales	Trametes_18663	Trametes_versicolor_281625
Basidiomycota	Polyporales	Junghuhnia_17873	Junghuhnia_nitida_315983
Basidiomycota	Polyporales	Mycoacia_18091	Mycoacia_fuscoatra_252296
Basidiomycota	Polyporales	Mycoacia_18091	Mycoacia_uda_255241
Basidiomycota	Polyporales	Irpex_17858	Irpex_lacteus_177211

Basidiomycota	Polyporales	Irpex_17858	pseudospecies_00168
Basidiomycota	Polyporales	Steccherinum_18582	Steccherinum_fimbriatum_306415
Basidiomycota	Polyporales	Steccherinum_18582	Steccherinum_ochraceum_122972
Basidiomycota	Polyporales	Gloeoporus_17670	pseudospecies_00201
Basidiomycota	Polyporales	Gloeoporus_17670	Gloeoporus_pannocinctus_297853
Basidiomycota	Polyporales	Scopuloides_18523	pseudospecies_00236
Basidiomycota	Polyporales	Scopuloides_18523	Scopuloides_rimosa_111015
Basidiomycota	Polyporales	Scopuloides_18523	Scopuloides_hydnoides_323312
Basidiomycota	Polyporales	Phlebia_18250	Phlebia_subserialis_302864
Basidiomycota	Polyporales	Phlebia_18250	Phlebia_acanthocystis_445231
Basidiomycota	Polyporales	Phlebia_18250	pseudospecies_00295
Basidiomycota	Polyporales	Phlebia_18250	Phlebia_tuberculata_518782
Basidiomycota	Polyporales	Phlebia_18250	Phlebia_radiata_204324
Basidiomycota	Polyporales	Bjerkandera_17160	Bjerkandera_adusta_100902
Basidiomycota	Trichosporonales	Trichosporon_10296	pseudospecies_00135
Basidiomycota	Cystofilobasidiales	pseudogenus_00037	pseudospecies_00131
Basidiomycota	Cystofilobasidiales	Udeniomyces_27406	Udeniomyces_pyricola_541906
Basidiomycota	Cystofilobasidiales	Mrakia_25264	Mrakia_aquatica_812175
Basidiomycota	Cystofilobasidiales	Cystofilobasidium_25635	Cystofilobasidium_infirmominiatum_135375
Basidiomycota	Cystofilobasidiales	Cystofilobasidium_25635	Cystofilobasidium_capitatum_106046
Basidiomycota	Cystofilobasidiales	Cystofilobasidium_25635	Cystofilobasidium_macerans_517356
Basidiomycota	Cystofilobasidiales	Itersonilia_8644	Itersonilia_perplexans_287352
Basidiomycota	Cystofilobasidiales	Itersonilia_8644	Itersonilia_pannonica_812189
Basidiomycota	Filobasidiales	Solicoccozyma_812197	pseudospecies_00614
Basidiomycota	Filobasidiales	Filobasidium_16130	Filobasidium_chernovii_812191
Basidiomycota	Filobasidiales	Filobasidium_16130	Filobasidium_oeirense_812193
Basidiomycota	Filobasidiales	Filobasidium_16130	Filobasidium_wieringae_812195
Basidiomycota	Tremellales	pseudogenus_00051	pseudospecies_00161
Basidiomycota	Tremellales	Hannaella_508459	pseudospecies_00233
Basidiomycota	Tremellales	Hannaella_508459	Hannaella_oryzae_508463
Basidiomycota	Tremellales	Hannaella_508459	Hannaella_sinensis_508464
Basidiomycota	Tremellales	Vishniacozyma_813272	Vishniacozyma_dimennae_813275
Basidiomycota	Tremellales	Vishniacozyma_813272	Vishniacozyma_globispora_813273
Basidiomycota	Tremellales	pseudogenus_00150	pseudospecies_01007
Basidiomycota	Tremellales	pseudogenus_00159	pseudospecies_00436
Basidiomycota	Tremellales	Naganishia_9074	pseudospecies_00350
Basidiomycota	Tremellales	Naganishia_9074	pseudospecies_00073
Basidiomycota	Tremellales	Naganishia_9074	pseudospecies_00060
Basidiomycota	Tremellales	Naganishia_9074	pseudospecies_00052
Basidiomycota	Tremellales	Papiliotrema_28647	Papiliotrema_fonsecae_813290
Basidiomycota	Tremellales	Bulleromyces_20056	Bulleromyces_albus_128947
Basidiomycota	Tremellales	Dioszegia_8042	pseudospecies_00512
Basidiomycota	Tremellales	Dioszegia_8042	Dioszegia_butyracea_506805
Basidiomycota	Tremellales	Dioszegia_8042	Dioszegia_catarinoi_521327
Basidiomycota	Tremellales	Dioszegia_8042	Dioszegia_fristingensis_521328
Basidiomycota	Tremellales	Dioszegia_8042	Dioszegia_aurantiaca_484349
Basidiomycota	Tremellales	Dioszegia_8042	Dioszegia_hungarica_296785
Basidiomycota	Tremellales	Cryptococcus_11060	pseudospecies_00187
Basidiomycota	Tremellales	Cryptococcus_11060	pseudospecies_00223
Basidiomycota	Tremellales	Cryptococcus_11060	Cryptococcus_laurentii_296158
Basidiomycota	Tremellales	Cryptococcus_11060	pseudospecies_00083
Basidiomycota	Tremellales	Cryptococcus_11060	pseudospecies_00180
Basidiomycota	Tremellales	Cryptococcus_11060	pseudospecies_00023
Basidiomycota	Tremellales	Cryptococcus_11060	pseudospecies_00007
Ascomycota	pseudoorder_0019	pseudogenus_00089	pseudospecies_00265
Ascomycota	pseudoorder_0009	pseudogenus_00034	pseudospecies_00354
Ascomycota	pseudoorder_0009	pseudogenus_00034	pseudospecies_00122
Ascomycota	dummy_order_Knufia	Knufia_27605	Knufia_petricola_804097
Ascomycota	Abrothallales	Phlebia_3	Phlebia_tremellosa_106356

Ascomycota	Teloschistales	Phaeophyscia_3939	Phaeophyscia_orbicularis_343218
Ascomycota	Teloschistales	Physcia_4082	Physcia_adscendens_400548
Ascomycota	Teloschistales	Physcia_4082	pseudospecies_00056
Ascomycota	Teloschistales	Physcia_4082	pseudospecies_00050
Ascomycota	Lecanorales	pseudogenus_00023	pseudospecies_00096
Ascomycota	Lecanorales	pseudogenus_00021	pseudospecies_00088
Ascomycota	Lecanorales	Melanelia_3061	Melanelia_subaurifera_342558
Ascomycota	Lecanorales	pseudogenus_00008	pseudospecies_00087
Ascomycota	Lecanorales	pseudogenus_00008	pseudospecies_00045
Ascomycota	Lecanorales	pseudogenus_00009	pseudospecies_00047
Ascomycota	Saccharomycetales	Clavispora_1095	Clavispora_lusitaniae_111257
Ascomycota	Saccharomycetales	Hyphopichia_2423	Hyphopichia_burtonii_315565
Ascomycota	Saccharomycetales	Meyerozyma_513456	Meyerozyma_guilliermondii_513463
Ascomycota	Saccharomycetales	Candida_7487	pseudospecies_00138
Ascomycota	Saccharomycetales	Candida_7487	Candida_sake_283382
Ascomycota	Saccharomycetales	Candida_7487	Candida_tropicalis_280770
Ascomycota	Saccharomycetales	Candida_7487	Candida_catenulata_284767
Ascomycota	Saccharomycetales	Dipodascus_1632	Dipodascus_geotrichum_313244
Ascomycota	Saccharomycetales	Saccharomyces_542401	Saccharomyces_cerevisiae_163963
Ascomycota	Saccharomycetales	Debaryomyces_1432	pseudospecies_00117
Ascomycota	Taphrinales	pseudogenus_00013	pseudospecies_00054
Ascomycota	Taphrinales	pseudogenus_00092	pseudospecies_00276
Ascomycota	Taphrinales	Taphrina_5354	Taphrina_padi_282516
Ascomycota	Taphrinales	Taphrina_5354	Taphrina_sacchari_277201
Ascomycota	Taphrinales	Taphrina_5354	pseudospecies_00082
Ascomycota	Taphrinales	Taphrina_5354	pseudospecies_00019
Ascomycota	Coniochaetales	Coniochaeta_1209	pseudospecies_01444
Ascomycota	Sordariales	Chaetomium_953	pseudospecies_01071
Ascomycota	Xylariales	Diatrype_1504	Diatrype_spilomea_254792
Ascomycota	Diaporthales	pseudogenus_00097	pseudospecies_00290
Ascomycota	Diaporthales	Cytospora_7904	pseudospecies_00311
Ascomycota	Diaporthales	Cytospora_7904	pseudospecies_00961
Ascomycota	dummy_order_Plectosphaerellaceae	Plectosphaerella_4197	Plectosphaerella_cucumerina_320609
Ascomycota	dummy_order_Myrmecridium	Myrmecridium_504559	Myrmecridium_schulzeri_504560
Ascomycota	Amphisphaeriales	pseudogenus_00078	pseudospecies_00251
Ascomycota	Amphisphaeriales	Pestalotiopsis_9272	pseudospecies_00277
Ascomycota	Amphisphaeriales	Pestalotiopsis_9272	pseudospecies_00235
Ascomycota	Amphisphaeriales	Microdochium_8926	Microdochium_bolleyi_317661
Ascomycota	Amphisphaeriales	Microdochium_8926	pseudospecies_00285
Ascomycota	Amphisphaeriales	Microdochium_8926	Microdochium_majus_345479
Ascomycota	Hypocreales	pseudogenus_00077	pseudospecies_00237
Ascomycota	Hypocreales	Beauveria_7346	pseudospecies_00077
Ascomycota	Hypocreales	Fusarium_8284	pseudospecies_00107
Ascomycota	Hypocreales	Fusarium_8284	Fusarium_poae_119380
Ascomycota	Hypocreales	pseudogenus_00020	pseudospecies_00080
Ascomycota	dummy_order_Nigrospora	Nigrospora_9124	pseudospecies_00055
Ascomycota	dummy_order_Coniosporium	Coniosporium_7755	Coniosporium_apollinis_444794
Ascomycota	dummy_order_Coniosporium	Coniosporium_7755	pseudospecies_01154
Ascomycota	Phaeomoniellales	Neophaeomoniella_812464	pseudospecies_00444
Ascomycota	Chaetothyriales	pseudogenus_00134	pseudospecies_00389
Ascomycota	Chaetothyriales	pseudogenus_00129	pseudospecies_00374
Ascomycota	Chaetothyriales	Neophaeococcomyces_814935	Neophaeococcomyces_aloes_814936
Ascomycota	Chaetothyriales	Strelitziana_501009	pseudospecies_00222
Ascomycota	Chaetothyriales	Bradymyces_808780	pseudospecies_00089
Ascomycota	Chaetothyriales	Metulocladosporiella_500224	pseudospecies_00163
Ascomycota	Chaetothyriales	Rhinocladiella_9720	pseudospecies_00653
Ascomycota	Chaetothyriales	Cladophialophora_7677	pseudospecies_00078
Ascomycota	Chaetothyriales	Cladophialophora_7677	pseudospecies_00043
Ascomycota	Chaetothyriales	Capronia_815	pseudospecies_00159

Ascomycota	Chaetothyriales	Capronia_815	pseudospecies_00982
Ascomycota	Chaetothyriales	Capronia_815	pseudospecies_00508
Ascomycota	Chaetothyriales	Capronia_815	pseudospecies_00456
Ascomycota	Chaetothyriales	Capronia_815	pseudospecies_00502
Ascomycota	Chaetothyriales	Capronia_815	pseudospecies_00074
Ascomycota	Chaetothyriales	Capronia_815	pseudospecies_00037
Ascomycota	Chaetothyriales	Capronia_815	pseudospecies_00017
Ascomycota	Eurotiales	Penicillium_9257	Penicillium_glaucoalbidum_561965
Ascomycota	Eurotiales	Penicillium_9257	pseudospecies_00155
Ascomycota	Eurotiales	Penicillium_9257	pseudospecies_00061
Ascomycota	Eurotiales	Penicillium_9257	pseudospecies_00485
Ascomycota	Eurotiales	Penicillium_9257	pseudospecies_00021
Ascomycota	Eurotiales	Aspergillus_7248	Aspergillus_penicillioides_309234
Ascomycota	Eurotiales	Aspergillus_7248	pseudospecies_00014
Ascomycota	Rhytismatales	Lophodermium_2941	Lophodermium_pinastri_212574
Ascomycota	Rhytismatales	Lophodermium_2941	Lophodermium_piceae_121134
Ascomycota	dummy_order_Myxotrichaceae	Pseudogymnoascus_4436	pseudospecies_00226
Ascomycota	pseudoorder_0005	pseudogenus_00133	pseudospecies_00388
Ascomycota	pseudoorder_0005	pseudogenus_00128	pseudospecies_00368
Ascomycota	pseudoorder_0005	pseudogenus_00015	pseudospecies_00062
Ascomycota	Thelebolales	Thelebolus_5419	pseudospecies_00101
Ascomycota	Erysiphales	Golovinomyces_25430	pseudospecies_00160
Ascomycota	Erysiphales	Golovinomyces_25430	pseudospecies_00224
Ascomycota	Erysiphales	Erysiphe_1898	pseudospecies_00227
Ascomycota	Erysiphales	Erysiphe_1898	pseudospecies_00113
Ascomycota	Erysiphales	Sawadaea_4873	Sawadaea_bicornis_258459
Ascomycota	Erysiphales	Podosphaera_4283	pseudospecies_00453
Ascomycota	Erysiphales	Podosphaera_4283	pseudospecies_00310
Ascomycota	Erysiphales	Podosphaera_4283	pseudospecies_00361
Ascomycota	Erysiphales	Podosphaera_4283	Podosphaera_leucotricha_122748
Ascomycota	Erysiphales	Blumeria_600	Blumeria_graminis_309596
Ascomycota	Helotiales	pseudogenus_00064	pseudospecies_00196
Ascomycota	Helotiales	pseudogenus_00169	pseudospecies_00467
Ascomycota	Helotiales	pseudogenus_00122	pseudospecies_00358
Ascomycota	Helotiales	pseudogenus_00095	pseudospecies_00286
Ascomycota	Helotiales	Tetracladium_10191	pseudospecies_00549
Ascomycota	Helotiales	Tetracladium_10191	pseudospecies_00532
Ascomycota	Helotiales	pseudogenus_00205	pseudospecies_01531
Ascomycota	Helotiales	Alatospora_7085	pseudospecies_00092
Ascomycota	Helotiales	pseudogenus_00412	pseudospecies_01082
Ascomycota	Helotiales	pseudogenus_00137	pseudospecies_00396
Ascomycota	Helotiales	pseudogenus_00250	pseudospecies_00652
Ascomycota	Helotiales	Mollisia_3239	pseudospecies_00253
Ascomycota	Helotiales	Mollisia_3239	pseudospecies_00162
Ascomycota	Helotiales	pseudogenus_00022	pseudospecies_00091
Ascomycota	Helotiales	Pyrenopeziza_4594	pseudospecies_00186
Ascomycota	Helotiales	Pyrenopeziza_4594	pseudospecies_00118
Ascomycota	Helotiales	pseudogenus_00070	pseudospecies_00207
Ascomycota	Helotiales	pseudogenus_00257	pseudospecies_00676
Ascomycota	Helotiales	pseudogenus_00031	pseudospecies_00111
Ascomycota	Helotiales	pseudogenus_00007	pseudospecies_00039
Ascomycota	Helotiales	pseudogenus_00005	pseudospecies_00033
Ascomycota	Helotiales	pseudogenus_00289	pseudospecies_00769
Ascomycota	Helotiales	pseudogenus_00042	pseudospecies_00137
Ascomycota	Helotiales	Crocicreas_1294	pseudospecies_01148
Ascomycota	Helotiales	Crocicreas_1294	pseudospecies_00556
Ascomycota	Helotiales	pseudogenus_00012	pseudospecies_00366
Ascomycota	Helotiales	pseudogenus_00012	pseudospecies_00053
Ascomycota	Helotiales	pseudogenus_00010	pseudospecies_00048

Ascomycota	Helotiales	pseudogenus_00047	pseudospecies_00148
Ascomycota	Helotiales	pseudogenus_00001	pseudospecies_00115
Ascomycota	Helotiales	pseudogenus_00001	pseudospecies_00006
Ascomycota	dummy_order_Pyrenochaeta	Pyrenochaeta_9667	Pyrenochaeta_cava_514652
Ascomycota	Venturiales	Venturia_5717	Venturia_inaequalis_164141
Ascomycota	Botryosphaeriales	pseudogenus_00234	pseudospecies_00831
Ascomycota	Botryosphaeriales	pseudogenus_00234	pseudospecies_00622
Ascomycota	Botryosphaeriales	Diplodia_8047	pseudospecies_00551
Ascomycota	Botryosphaeriales	Lasiodiplodia_8708	pseudospecies_00123
Ascomycota	Botryosphaeriales	pseudogenus_00176	pseudospecies_00482
Ascomycota	Botryosphaeriales	pseudogenus_00085	pseudospecies_00255
Ascomycota	Botryosphaeriales	pseudogenus_00145	pseudospecies_00412
Ascomycota	Botryosphaeriales	pseudogenus_00088	pseudospecies_00264
Ascomycota	Botryosphaeriales	pseudogenus_00127	pseudospecies_00367
Ascomycota	dummy_order_Leptospora	Leptospora_2803	pseudospecies_00188
Ascomycota	dummy_order_Leptospora	Leptospora_2803	pseudospecies_00156
Ascomycota	dummy_order_Leptospora	Leptospora_2803	pseudospecies_00051
Ascomycota	dummy_order_Leptospora	Leptospora_2803	Leptospora_rubella_119461
Ascomycota	pseudoorder_0006	pseudogenus_00068	pseudospecies_00203
Ascomycota	pseudoorder_0006	pseudogenus_00024	pseudospecies_00202
Ascomycota	pseudoorder_0006	pseudogenus_00024	pseudospecies_00097
Ascomycota	pseudoorder_0006	pseudogenus_00016	pseudospecies_00067
Ascomycota	Dothideales	pseudogenus_00115	pseudospecies_00337
Ascomycota	Dothideales	pseudogenus_00086	pseudospecies_00256
Ascomycota	Dothideales	pseudogenus_00011	pseudospecies_00049
Ascomycota	Dothideales	Sydowia_5311	Sydowia_polyspora_306575
Ascomycota	Dothideales	pseudogenus_00014	pseudospecies_00057
Ascomycota	Dothideales	pseudogenus_00123	pseudospecies_00359
Ascomycota	Dothideales	pseudogenus_00409	pseudospecies_01072
Ascomycota	Dothideales	Aureobasidium_7297	Aureobasidium_thailandense_801148
Ascomycota	Dothideales	Aureobasidium_7297	pseudospecies_00004
Ascomycota	Pleosporales	Cucurbitaria_1348	pseudospecies_00558
Ascomycota	Pleosporales	Lophiostoma_2933	Lophiostoma_corticola_483959
Ascomycota	Pleosporales	pseudogenus_00450	pseudospecies_01192
Ascomycota	Pleosporales	Corynespora_7795	Corynespora_cassiicola_296024
Ascomycota	Pleosporales	pseudogenus_00184	pseudospecies_00505
Ascomycota	Pleosporales	pseudogenus_00046	pseudospecies_00145
Ascomycota	Pleosporales	pseudogenus_00026	pseudospecies_00175
Ascomycota	Pleosporales	pseudogenus_00026	pseudospecies_00104
Ascomycota	Pleosporales	Leptosphaeria_2800	pseudospecies_00590
Ascomycota	Pleosporales	Leptosphaeria_2800	pseudospecies_00387
Ascomycota	Pleosporales	Leptosphaeria_2800	pseudospecies_00108
Ascomycota	Pleosporales	Leptosphaeria_2800	pseudospecies_00149
Ascomycota	Pleosporales	Leptosphaeria_2800	pseudospecies_00090
Ascomycota	Pleosporales	pseudogenus_00036	pseudospecies_00130
Ascomycota	Pleosporales	Phaeosphaeria_3951	pseudospecies_00151
Ascomycota	Pleosporales	Phaeosphaeria_3951	pseudospecies_00141
Ascomycota	Pleosporales	Phaeosphaeria_3951	pseudospecies_00116
Ascomycota	Pleosporales	Phaeosphaeria_3951	pseudospecies_00084
Ascomycota	Pleosporales	Phaeosphaeria_3951	pseudospecies_00075
Ascomycota	Pleosporales	Phaeosphaeria_3951	pseudospecies_00071
Ascomycota	Pleosporales	Phaeosphaeria_3951	pseudospecies_00038
Ascomycota	Pleosporales	Phaeosphaeria_3951	pseudospecies_00031
Ascomycota	Pleosporales	Periconia_9263	pseudospecies_00126
Ascomycota	Pleosporales	Periconia_9263	pseudospecies_00164
Ascomycota	Pleosporales	Periconia_9263	Periconia_byssoides_144538
Ascomycota	Pleosporales	Edenia_510872	Edenia_gomezpompae_510944
Ascomycota	Pleosporales	Curvularia_7847	Curvularia_inaequalis_267748
Ascomycota	Pleosporales	Curvularia_7847	pseudospecies_00195

Ascomycota	Pleosporales	Curvularia_7847	pseudospecies_00150
Ascomycota	Pleosporales	Curvularia_7847	pseudospecies_00382
Ascomycota	Pleosporales	Curvularia_7847	pseudospecies_00112
Ascomycota	Pleosporales	Dendryphion_7952	Dendryphion_nanum_296539
Ascomycota	Pleosporales	Pyrenophora_4596	pseudospecies_00657
Ascomycota	Pleosporales	Pyrenophora_4596	Pyrenophora_tritici-repentis_255190
Ascomycota	Pleosporales	pseudogenus_00019	pseudospecies_00103
Ascomycota	Pleosporales	pseudogenus_00019	pseudospecies_00070
Ascomycota	Pleosporales	pseudogenus_00017	pseudospecies_00068
Ascomycota	Pleosporales	Bipolaris_7375	pseudospecies_00173
Ascomycota	Pleosporales	Bipolaris_7375	pseudospecies_00072
Ascomycota	Pleosporales	Pleospora_4233	pseudospecies_00576
Ascomycota	Pleosporales	Pleospora_4233	pseudospecies_00011
Ascomycota	Pleosporales	Alternaria_7106	pseudospecies_00303
Ascomycota	Pleosporales	Alternaria_7106	Alternaria_brassicae_214057
Ascomycota	Pleosporales	Alternaria_7106	pseudospecies_00026
Ascomycota	Pleosporales	Alternaria_7106	pseudospecies_00064
Ascomycota	Pleosporales	Alternaria_7106	pseudospecies_00009
Ascomycota	Pleosporales	Alternaria_7106	pseudospecies_00003
Ascomycota	Pleosporales	Phoma_9358	pseudospecies_00121
Ascomycota	Pleosporales	Phoma_9358	pseudospecies_00385
Ascomycota	Pleosporales	Phoma_9358	pseudospecies_00144
Ascomycota	Pleosporales	Phoma_9358	pseudospecies_00008
Ascomycota	Pleosporales	Ascochyta_7239	pseudospecies_00309
Ascomycota	Pleosporales	Ascochyta_7239	pseudospecies_00086
Ascomycota	Pleosporales	Ascochyta_7239	pseudospecies_00079
Ascomycota	Pleosporales	Ascochyta_7239	pseudospecies_00100
Ascomycota	Pleosporales	Ascochyta_7239	pseudospecies_00146
Ascomycota	Pleosporales	Ascochyta_7239	pseudospecies_00042
Ascomycota	Pleosporales	Ascochyta_7239	pseudospecies_00005
Ascomycota	Capnodiales	pseudogenus_00223	pseudospecies_00598
Ascomycota	Capnodiales	pseudogenus_00185	pseudospecies_00507
Ascomycota	Capnodiales	pseudogenus_00104	pseudospecies_00305
Ascomycota	Capnodiales	pseudogenus_00107	pseudospecies_00853
Ascomycota	Capnodiales	pseudogenus_00033	pseudospecies_00120
Ascomycota	Capnodiales	Dissoconium_11074	Dissoconium_proteae_506593
Ascomycota	Capnodiales	pseudogenus_00028	pseudospecies_00106
Ascomycota	Capnodiales	Torula_10248	pseudospecies_00125
Ascomycota	Capnodiales	Torula_10248	Torula_herbarum_199478
Ascomycota	Capnodiales	pseudogenus_00079	pseudospecies_00241
Ascomycota	Capnodiales	pseudogenus_00074	pseudospecies_00213
Ascomycota	Capnodiales	pseudogenus_00194	pseudospecies_00530
Ascomycota	Capnodiales	pseudogenus_00027	pseudospecies_00105
Ascomycota	Capnodiales	Neocatenulostroma_807805	pseudospecies_00142
Ascomycota	Capnodiales	pseudogenus_00065	pseudospecies_00198
Ascomycota	Capnodiales	pseudogenus_00043	pseudospecies_00139
Ascomycota	Capnodiales	pseudogenus_00057	pseudospecies_00178
Ascomycota	Capnodiales	Rachicladosporium_504430	pseudospecies_00675
Ascomycota	Capnodiales	pseudogenus_00060	pseudospecies_00183
Ascomycota	Capnodiales	pseudogenus_00052	pseudospecies_00165
Ascomycota	Capnodiales	pseudogenus_00049	pseudospecies_00154
Ascomycota	Capnodiales	pseudogenus_00058	pseudospecies_00179
Ascomycota	Capnodiales	pseudogenus_00119	pseudospecies_00352
Ascomycota	Capnodiales	pseudogenus_00030	pseudospecies_00110
Ascomycota	Capnodiales	pseudogenus_00030	pseudospecies_00174
Ascomycota	Capnodiales	pseudogenus_00018	pseudospecies_00069
Ascomycota	Capnodiales	Pseudocercospora_9559	pseudospecies_00259
Ascomycota	Capnodiales	Pseudocercospora_9559	pseudospecies_00076
Ascomycota	Capnodiales	Pseudocercospora_9559	pseudospecies_00040

Ascomycota	Capnodiales	Ramularia_9691	pseudospecies_00066
Ascomycota	Capnodiales	Ramularia_9691	pseudospecies_00093
Ascomycota	Capnodiales	Cladosporium_7681	pseudospecies_00129
Ascomycota	Capnodiales	Cladosporium_7681	pseudospecies_00099
Ascomycota	Capnodiales	Cladosporium_7681	pseudospecies_00098
Ascomycota	Capnodiales	Cladosporium_7681	pseudospecies_00095
Ascomycota	Capnodiales	Cladosporium_7681	pseudospecies_00041
Ascomycota	Capnodiales	Cladosporium_7681	pseudospecies_00035
Ascomycota	Capnodiales	Cladosporium_7681	pseudospecies_00032
Ascomycota	Capnodiales	Cladosporium_7681	pseudospecies_00020
Ascomycota	Capnodiales	Cladosporium_7681	pseudospecies_00018
Ascomycota	Capnodiales	Cladosporium_7681	pseudospecies_00016
Ascomycota	Capnodiales	Cladosporium_7681	pseudospecies_00015
Ascomycota	Capnodiales	Cladosporium_7681	pseudospecies_00013
Ascomycota	Capnodiales	Cladosporium_7681	pseudospecies_00012
Ascomycota	Capnodiales	Cladosporium_7681	pseudospecies_00010
Ascomycota	Capnodiales	Cladosporium_7681	pseudospecies_00002
Ascomycota	Capnodiales	Cladosporium_7681	pseudospecies_00001

Table S5. AIC-based comparison between univariate models fitted to species richness, with data contaminated with sequences counts observed in negative controls. The AIC-values are shown for the climatic models CS1-CS4 as well as for the best supported weather model variant of the model CS4 (W.best). The values are shown relative to the AIC value of model CS1. The three numbers in each cell, from up to down, show the mean, minimum and maximum values over the ten replicates of the contaminated datasets. For comparison with the original data, see Table S1.

Model/Response	CS1	CS2	CS3	CS4	W.best
Overall diversity					
Species richness	0<br>0<br>0	4.35<br>3.90<br>5.02	-227.83<br>-238.13<br>-216.29	-419.39<br>-429.33<br>-404.50	-468.11<br>-475.24<br>-452.76
Trophic guild-specific species richness					
Plant pathogen	0<br>0<br>0	6.89<br>6.59<br>7.51	-256.71<br>-266.31<br>-246.64	-479.93<br>-481.10<br>-455.46	-510.19<br>-520.94<br>-495.33
Saprotroph	0<br>0<br>0	8.70<br>8.43<br>9.19	-221.87<br>-230.13<br>-212.47	-411.67<br>-420.80<br>-397.02	-451.78<br>-460.17<br>-437.85
Wood saprotroph	0<br>0<br>0	1.31<br>1.01<br>1.64	-249.99<br>-254.22<br>-245.53	-447.01<br>-455.07<br>-437.83	-490.47<br>-498.85<br>-482.19
Endophyte	0<br>0<br>0	10.78<br>10.37<br>11.27	-238.87<br>-243.96<br>-230.96	-459.67<br>-468.47<br>-446.94	-503.70<br>-513.09<br>-493.43
Ectomycorrhizal	0<br>0<br>0	24.76<br>24.58<br>25.07	-100.41<br>-103.28<br>-97.22	-182.89<br>-186.55<br>-177.42	-206.22<br>-209.15<br>-200.98
Animal pathogen	0<br>0<br>0	11.35<br>10.97<br>11.99	-192.77<br>-200.03<br>-183.38	-421.58<br>-431.95<br>-441.49	-458.87<br>-467.71<br>-448.69
Lichenized	0<br>0<br>0	17.99<br>17.98<br>18.03	-117.71<br>-117.78<br>-117.62	-243.49<br>-243.62<br>-243.35	-302.53<br>-302.69<br>-302.36
Epiphyte	0<br>0<br>0	14.37<br>14.12<br>14.82	-180.59<br>-192.50<br>-169.75	-418.68<br>-425.83<br>-405.27	-453.86<br>-460.04<br>-440.24
Ericoid mycorrhizal	0<br>0<br>0	21.78<br>21.77<br>21.80	-131.08<br>-131.17<br>-130.97	-254.06<br>-254.23<br>-253.90	-272.74<br>-272.91<br>-272.56

Table S6. R^2-based comparison between univariate models fitted to species richness, with data contaminated with sequences counts observed in negative controls. The R^2-values are shown for the climatic models CS1-CS4 as well as for the best supported weather model variant of the model CS4 (W.best) The R^2-values were computed with the r2-function of the R-package sjstats[2]. The three numbers in each cell, from up to down, show the mean, minimum and maximum values over the ten replicates of the contaminated datasets. For comparison with the original data, see Table S2.

Model/Response	CS1	CS2	CS3	CS4	W.best
Overall diversity					
Species richness	0.30<br>0.29<br>0.30	0.29<br>0.29<br>0.30	0.36<br>0.36<br>0.37	0.38<br>0.37<br>0.39	0.40<br>0.39<br>0.40
Trophic guild-specific species richness					
Plant pathogen	0.30<br>0.29<br>0.30	0.29<br>0.29<br>0.30	0.37<br>0.37<br>0.38	0.39<br>0.39<br>0.40	0.41<br>0.40<br>0.42
Saprotroph	0.30<br>0.29<br>0.30	0.30<br>0.29<br>0.30	0.36<br>0.36<br>0.37	0.39<br>0.38<br>0.39	0.40<br>0.39<br>0.41
Wood saprotroph	0.33<br>0.32<br>0.33	0.32<br>0.31<br>0.32	0.39<br>0.39<br>0.40	0.42<br>0.42<br>0.43	0.44<br>0.43<br>0.44
Endophyte	0.31<br>0.31<br>0.31	0.31<br>0.30<br>0.31	0.38<br>0.38<br>0.39	0.40<br>0.40<br>0.41	0.42<br>0.41<br>0.42
Ectomycorrhizal	0.16<br>0.16<br>0.16	0.16<br>0.16<br>0.16	0.21<br>0.21<br>0.21	0.22<br>0.22<br>0.23	0.24<br>0.24<br>0.24
Animal pathogen	0.31<br>0.31<br>0.32	0.31<br>0.31<br>0.32	0.37<br>0.36<br>0.38	0.39<br>0.39<br>0.40	0.41<br>0.40<br>0.41
Lichenized	0.19<br>0.19<br>0.19	0.20<br>0.20<br>0.20	0.25<br>0.25<br>0.25	0.25<br>0.25<br>0.25	0.27<br>0.27<br>0.27
Epiphyte	0.30<br>0.30<br>0.31	0.30<br>0.30<br>0.31	0.36<br>0.36<br>0.37	0.39<br>0.38<br>0.39	0.40<br>0.39<br>0.40
Ericoid mycorrhizal	0.14<br>0.14<br>0.14	0.15<br>0.15<br>0.15	0.21<br>0.21<br>0.21	0.21<br>0.21<br>0.21	0.23<br>0.23<br>0.23

Table S7. Results from the fitted weather models, with data contaminated with negative controls. The cells for the rows *Temperature*, *Precipitation,* and *Wind* indicate the effect sign ("+" for positive and "-" for negative) and temporal scale at which the weather covariate was included in the best supported weather model, with an empty cell indicating that the weather covariate was excluded from the model. The numbers in brackets describe the number of contaminated datasets (out of ten replicates) that resulted in the particular outcome. In case of discrepancy among the replicates, the mode is shown for the best weather model. For comparison with the original data, see Table S3.

Variable/Response	Tempe-rature	Precipi-tation	Wind	Best weather model
Overall diversity				
Species richness	1 (10)	0 (10)	1 (10)	W1_temp:day_prec:no_wind:day
Trophic guild-specific species richness				
Plant pathogen	1 (10)	0 (6)<br>-1 (4)	1 (10)	W1_temp:day_prec:no_wind:day
Saprotroph	1 (10)	0 (10)	1 (10)	W1_temp:day_prec:no_wind:day
Wood saprotroph	1 (10)	0 (10)	0 (10)	W1_temp:day_prec:no_wind:no
Endophyte	1 (10)	-1 (9)<br>0 (1)	1 (10)	W1_temp:day_prec:week_wind:day
Ectomycorrhizal	1 (10)	0 (10)	1 (7)<br>0 (3)	W1_temp:day_prec:no_wind:no
Animal pathogen	1 (10)	0 (10)	1 (10)	W1_temp:day_prec:no_wind:day
Lichenized	1 (10)	-1 (10)	1 (10)	W1_temp:day_prec:day_wind:day
Epiphyte	1 (10)	0 (10)	1 (10)	W1_temp:day_prec:no_wind:day
Ericoid mycorrhizal	1 (10)	0 (10)	0 (10)	W2_temp:day_prec:no_wind:no
